# Intracellular RNA and DNA tracking by uridine-rich internal loop tagging with fluorogenic bPNA

**DOI:** 10.1038/s41467-023-38579-2

**Published:** 2023-05-24

**Authors:** Yufeng Liang, Sydney Willey, Yu-Chieh Chung, Yi-Meng Lo, Shiqin Miao, Sarah Rundell, Li-Chun Tu, Dennis Bong

**Affiliations:** 1 Department of Chemistry & Biochemistry, The Ohio State University, Columbus, OH USA; 2 Center for RNA Biology, The Ohio State University, Columbus, OH USA; 3 Department of Biological Chemistry and Pharmacology, The Ohio State University, Columbus, OH USA; 4 The Ohio State University Comprehensive Cancer Center, The Ohio State University, Columbus, OH USA

**Keywords:** RNA, Biosensors, Chromosomes

## Abstract

The most widely used method for intracellular RNA fluorescence labeling is MS2 labeling, which generally relies on the use of multiple protein labels targeted to multiple RNA (MS2) hairpin structures installed on the RNA of interest (ROI). While effective and conveniently applied in cell biology labs, the protein labels add significant mass to the bound RNA, which potentially impacts steric accessibility and native RNA biology. We have previously demonstrated that internal, genetically encoded, uridine-rich internal loops (URILs) comprised of four contiguous UU pairs (8 nt) in RNA may be targeted with minimal structural perturbation by triplex hybridization with 1 kD bifacial peptide nucleic acids (bPNAs). A URIL-targeting strategy for RNA and DNA tracking would avoid the use of cumbersome protein fusion labels and minimize structural alterations to the RNA of interest. Here we show that URIL-targeting fluorogenic bPNA probes in cell media can penetrate cell membranes and effectively label RNAs and RNPs in fixed and live cells. This method, which we call fluorogenic U-rich internal loop (FLURIL) tagging, was internally validated through the use of RNAs bearing both URIL and MS2 labeling sites. Notably, a direct comparison of CRISPR-dCas labeled genomic loci in live U2OS cells revealed that FLURIL-tagged gRNA yielded loci with signal to background up to 7X greater than loci targeted by guide RNA modified with an array of eight MS2 hairpins. Together, these data show that FLURIL tagging provides a versatile scope of intracellular RNA and DNA tracking while maintaining a light molecular footprint and compatibility with existing methods.

## Abstract (web summary)

Commonly used protein-based tools to monitor intracellular RNA and DNA can impact steric accessibility and native nucleic acid biology. Here, the authors show that fluorogenic uridine-rich internal loop tagging bPNA probes can be used to label nucleic acids in fixed and live cells.

## Introduction

There remains a need for improved and orthogonal methods for localizing and tracking intracellular RNA and DNA molecules in real time^1^. Despite the existence of many useful RNA imaging strategies, there are limitations on the placement of probe binding sites and concerns regarding the overall structural impact of labeling on the RNA of interest. Modification of internal sites in RNA via a chemical reaction or duplex hybridization can be technically challenging or sequence-limited^2,3^. While fluorescence in situ (duplex) hybridization^4,5^ (FISH) with DNA or PNA probes is broadly used for labeling RNAs, FISH is limited to fixed cell experiments. Molecular beacons may be used with unmodified cells and transcripts in live cell labeling but require electroporation or microinjection strategies and have not been as widely adopted as other methods^1,6^. In addition, duplex hybridization to internal sites destroys existing secondary structures via strand invasion or transforms a single-stranded loop into a new duplex stem where none existed previously^4^, introducing potentially disruptive steric interactions. Beyond chemical modification and FISH, approaches to intracellular RNA tracking may be roughly divided into small molecule dye binding and protein labels. Dye-binding SELEX-derived aptamers (e.g., Spinach^7^, Broccoli^8^, Corn^9^, Mango^10^, Pepper^11^) have been inserted into RNAs of interest for tracking and sensing applications^12,13^. A variation on this approach exploits RNA binding to dequench dye-quencher conjugates (e.g., SRB2^14^, Gemini-561^15^, Riboglow^16^) and can utilize native aptamers to universal quenchers such as cobalamin^16^; this method may be applied to a wider range of fluorophores without the need for additional aptamer selection. However, all aptamer methods require the insertion of a distinct folded structure within the ROI that has the potential to alter RNA sterics or lifetime. Further, early fluorescent aptamer designs suffered from insufficient brightness, thought to be due to misfolded RNA loops^17^. In particular, the G-quadruplex motif common to Mango and Spinach aptamers^18–20^ is especially labile to intracellular degradation^21^ and requires non-native levels of potassium and magnesium^8^, which may result in deficient intracellular aptamer performance^1^. Bacteriophage-derived RNA hairpins MS2 and PP7 are native sequences that bind to phage coat proteins MCP (MS2 coat protein) and PCP (PP7 coat protein), respectively. MS2-labeling requires insertion of multiple MS2 hairpins into ROIs and with subsequent RNP imaging via binding of MCP fusion proteins that facilitate fluorescent readout (eg.- fluorescent proteins^22,23^ (MCP-FPs), MCP-HaloTag^24^ and MCP-SNAP tag^11,25^ fusions). Though effective, the use of constitutively fluorescent dyes and FPs generates a significant background signal, requiring multiple (often >20) MS2 hairpins to generate a sufficient signal to noise. Further, these protein labeling systems carry significant steric bulk (>40 kD for a single MCP-FP) with the risk of altered transcript decay^22,26–28^ and inhibited native contacts. Despite these drawbacks, MS2/PP7 labeling remains the most widely used method for RNA tracking. Similarly, CRISPR-Cas/guide RNA complexes^29^ can be used to track of DNA and RNA targets via fluorescent readout of protein fusions to Cas variants, but also via formation of sterically encumbering protein binding to native RNA^30^ targets or genomic loci^31^.

**Fig. 1:**
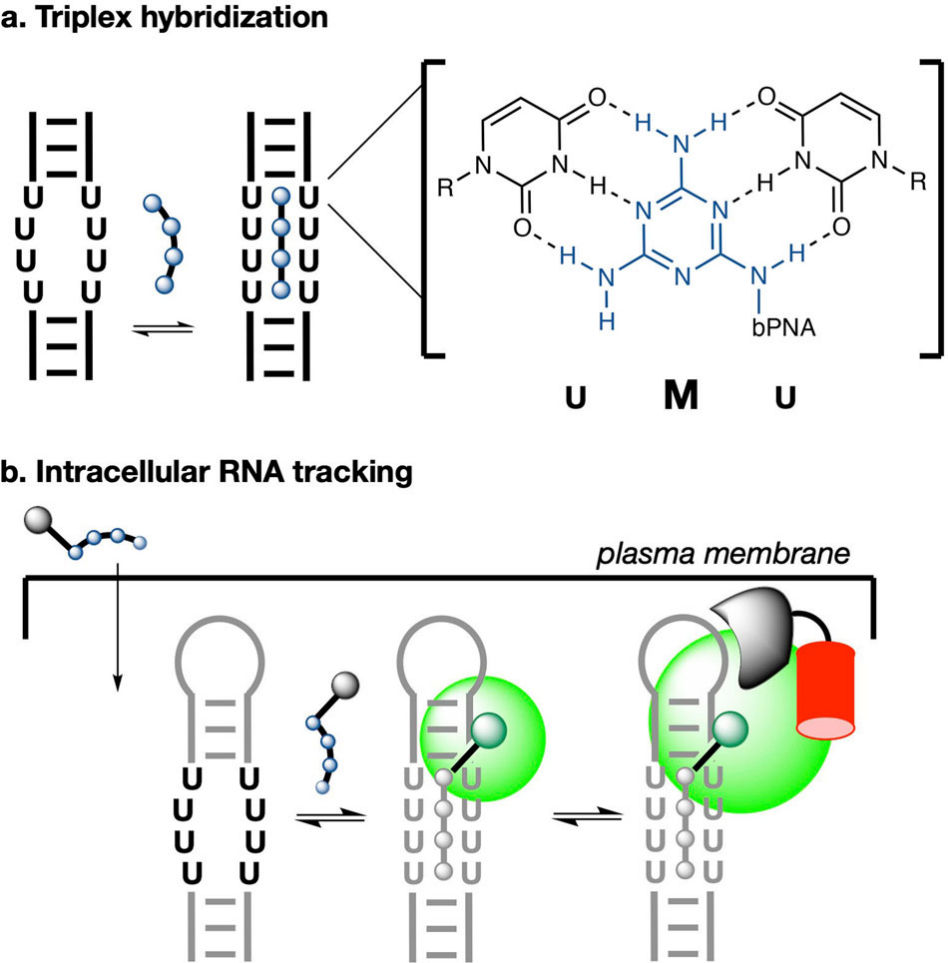
FLURIL tagging of RNAs with bPNA probes. **a** Triplex hybridization of a U-rich internal loop (URIL) with bPNA (blue) via base triple formation between the melamine base (M) and two uracil bases (inset). **b** General schematic of labeling strategy described herein. An RNA of interest is engineered to contain a URIL and expressed within the cell, with a fluorogenic bPNA probe introduced via cell culture media. Successful URIL targeting is reported by an increase in emission (green) and confirmed by colocalization with an RBP fusion with a (red) fluorescent protein.

**Fig. 2:**
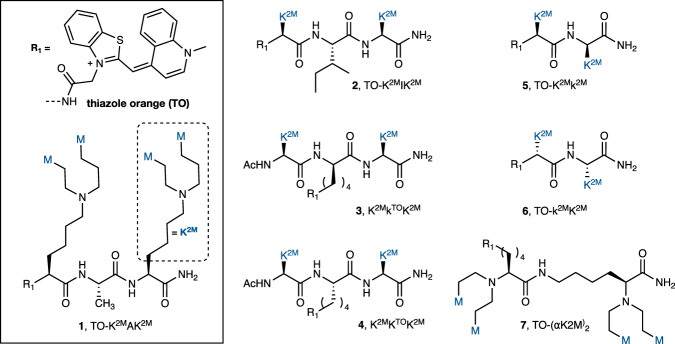
Synthetic bPNA probes. Structures of fluorogenic bPNA probes studied, with thiazole orange (TO) dye and K^2M^ sidechain structure shown inset. Lower case k^2M^ residue denotes D-configuration, and K^TO^ indicates sidechain is acylated with R_1_ (TO). For TO-K^2M^AK^2M^ and TO-K^2M^IK^2M^, R_1_ includes a β-alanine spacer between TO and bPNA.

Here, in a method we call fluorogenic U-rich internal loop (FLURIL) tagging, we show that intracellular RNAs bearing an 8-nt (U_4_xU_4_) U-rich internal loop (URIL) can be selectively labeled with a fluorogenic bPNA probe via triplex hybridization. The FLURIL system minimizes the introduction of new RNA secondary structures by use of duplex-for-triplex stem replacement, is considerably smaller than typical RNA aptamer or protein labeling systems, and exhibits improved signal-to-background and stability over comparable methods (Supplementary Fig. 3). Prior studies on triazine assembly^32–35^ and targeting^36,37^ led to our development of bifacial peptide nucleic acid (bPNA)^34,38,39^, which presents synthetic melamine bases^40,41^ on an α-peptide backbone^42–44^ (Fig. 1). The bPNA family of compounds selectively hybridize with U_n_xU_n_ internal bulges^38,39,45,46^ via formation of uracil-melamine-uracil (UMU) base triples, generating triplex stems that can functionally replace native RNA stems^45–47^, tertiary contacts^46,47^, block protein readthrough^48^, direct chemistry^49^, and modulate lncRNA lifetime^50^. Optimized^51^ cationic 4 M bPNAs (with four melamine bases, Fig. 2) are cell-permeable^49,50^ and can bind to structured U_4_xU_4_ loops (URILs) while retaining nanomolar affinity^45^, thus enabling specific intracellular bPNA targeting of URIL-tagged ROIs. via formation of triplex hybrid  stems that are similar in mass to a 4 bp RNA duplex. Via URIL-tagging, bPNA hybridization can place prosthetic groups at internal RNA sites without aptamer selection; this manuscript describes  fluorogen-modified bPNA binding to the URIL (FLURIL) tag. We demonstrate FLURIL tagging of intracellular RNAs and RNPs in both fixed and live cell contexts.

## Results

### Design and synthesis of fluorogenic bPNA probes for URIL RNAs

We previously reported that cyanine dye (Cy3, Cy5) modified bPNAs exhibited fluorescence enhancement upon triplex hybridization to URIL RNA and subsequent hybrid RNA-protein binding^45^. This was expected^52^ based on prior observations with DNA binding^53^. Thiazole orange dye is a more typical choice to signal intercalative binding due to its high emission in the bound state and low emission in the unbound state: Seitz and others^54^ have demonstrated sequence-selective “forced intercalation” (FIT) emission with PNA-thiazole orange (TO) conjugates^55^ that place TO at the hybridization interface. Similarly, Dervan has attached TO to pyrrole-imidazole polyamide (Py-Im) minor groove intercalators that fluoresce upon sequence recognition^56^. While these probes have utility, they are limited with regard to transport and RNA binding^57^. To study fluorogenic RNA binding in the context of URIL triplex hybridization, we synthesized bPNAs *N*-terminated with Cy5, difluorohydroxybenzylidene (DFHBI)^7^ and TO derivatives (Fig. 2 and Supplementary Fig. 18). Prior work has established that compact dipeptide or tripeptide bPNA scaffolds containing just four melamine base-tripling units (4 M bPNAs) can exhibit high affinity binding to a structured U_4_xU_4_ internal loop (URIL). Structure-function studies^51,58,59^ indicated that presentation of two melamine bases on the ε-amine of lysine (K^2M^) results in a solubilizing cationic sidechain that enables strong URIL binding even with short, low molecular weight di and tri-peptides. Even following *N*-terminal modification with fluorogenic modules, these efficient bPNA probes are in a low molecular weight regime (~1 kD). Moreover, Fmoc-K^2M^ can be obtained on a multigram scale by double reductive alkylation of Fmoc-lysine with melamine acetaldehyde without column purification^60^, making these bPNA probes are highly accessible and synthetically scalable. We prepared a small family of dye-modified (Cy3, Cy5, DFHBI, TO) 4 M bPNAs (Fig. 2), focusing primarily on the tripeptide (K^2M^X-K^2M^) scaffold where X is an α-amino acid and dipeptide isomers. Fluorogenic turn-on upon RNA complexation was modest for the cyanine and DFHBI dyes (Supplementary Figs. 7 and 14); we thus focused on TO derivatives. In K^2M^X-K^2M^, thiazole orange carboxylic acid was coupled via a β-alanine linker to the *N*-terminus of (X=Ala, Ile) or directly to the ε-amine of lysine (X = K^TO^). As dipeptide and tripeptide bPNAs of alternating amino acid configuration had previously shown an advantage in triplex hybridization over the homochiral dipeptides due to syndiotactic base presentation^51,61,62^, we set out to test the impact of stereochemistry on the fluorogenic binding when modified with TO on the *N*-terminus (TO-k^2M^K^2M^, TO-K^2M^k^2M^) and ε-amine of the central amino acid (K^2M^k^TO^-K^2M^). Additionally, we synthesized and evaluated the isodipeptide (TO-(αK^2M^)_2_) that features a sidechain linkage, with base presentation on the α-nitrogens and TO modification on the ε-amine.

### In vitro evaluation of fluorogenic URIL-RNA binding by bPNA variants

**Fig. 3:**
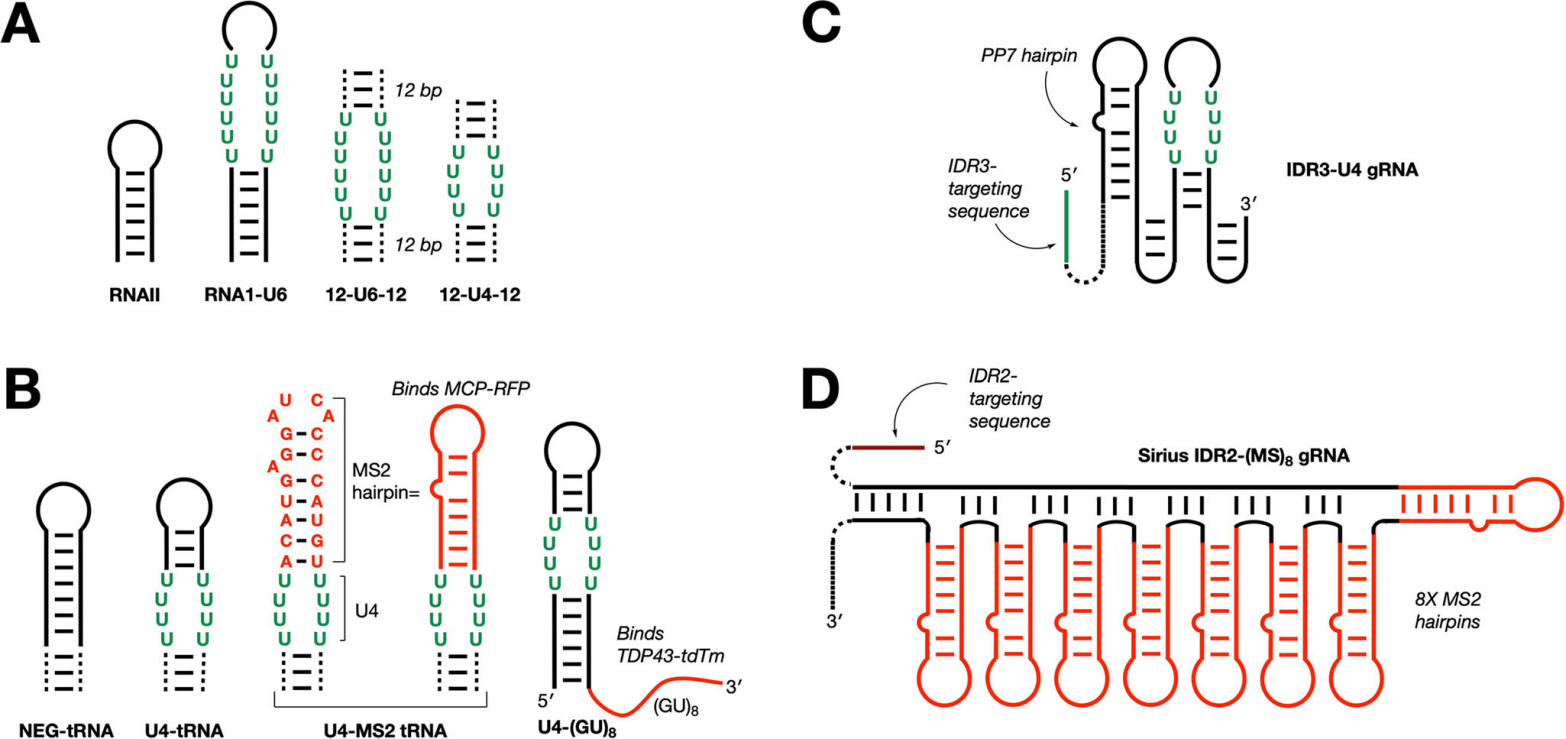
Design of URIL RNA constructs for bPNA probe binding. **A** RNAs for in vitro evaluation were prepared via run-off transcription or purchased directly. The 12-U6-12 and 12-U4-12 constructs feature 12 bp duplexes (indicated by dashed lines) separated by U_6_xU_6_ and U_4_xU_4_ internal bulges, respectively. **B** RNA constructs for fixed cell labeling (HEK-293T) of RNPs are shown; hairpin RNAs were used to replace the anticodon stem of tRNA^Lys^ (dashed lines) or appended to a TDP-43 binding (GU)_8_ sequence. Constructs for live cell (U2OS) RNA tracking with **C** FLURIL tagging and **D** Sirius-IDR2-(MS2)_8_ with MS2 (MBSV5)^84^ hairpins are shown in red. IDR3-U4 carries a single PP7 hairpin as an internal control. RNAs for cell experiments were delivered by plasmid transfection. See “Methods” and Supplementary Fig. 2 for sequences and protocols.

**Fig. 4:**
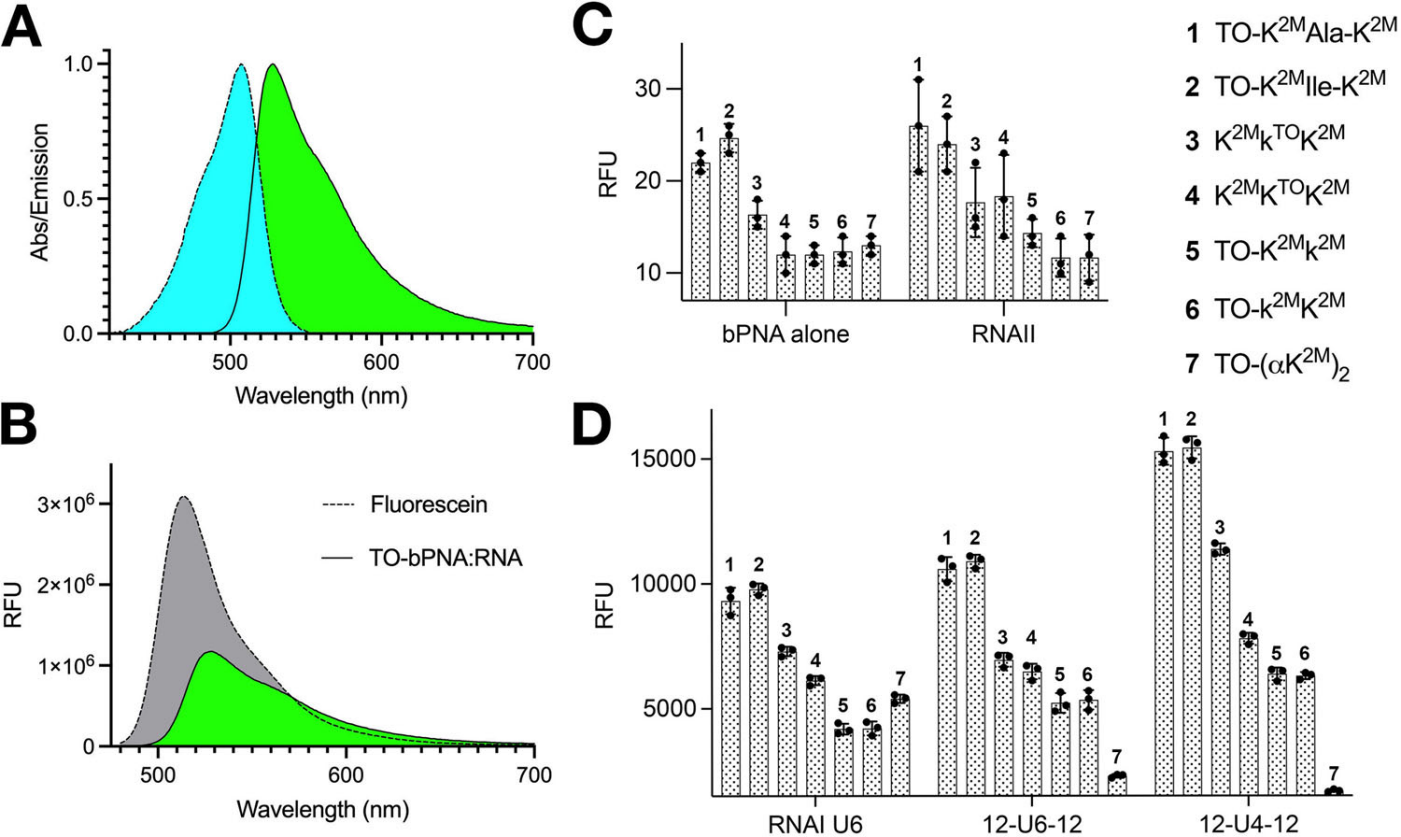
Characterization of fluorogenic bPNA hybridization with URIL RNAs. **A** Normalized absorbance and emission of a representative TO-bPNA **1** hybrid with RNA. **B** Relative fluorescence units (RFU) of fluorescein standard and the hybrid in (**A**) indicating relative brightness (Φ = 43%, ε_507_ = 44,883 M^−1^cm^−1^). In vitro fluorescence of **C** bPNAs alone or with RNAII (lacking a URIL) and **D** upon treatment with RNA constructs that have a URIL binding site (Fig. 2). The mean value of three separate measurements with standard deviation error for all RNA and bPNA combinations are shown under identical conditions (50 mM HEPES, pH 7.5, 100 mM NaCl, 1 µM bPNA and RNA).

With URIL-binding bPNAs in hand, we tested fluorogenic binding in vitro to hairpin and duplex URIL-RNAs (Fig. 3A). The absorbance and emission properties of the free TO-bPNAs were similar to the parent thiazole orange dye, which exhibits weak emission in solution (quantum yield 10^−4^)^63^. However, when TO-K^2M^Ala-K^2M^ binds to URIL RNAs (Fig. 3A), emission increases by up to 600-fold (Fig. 4, Supplementary Figs. 12 and 13). Indeed, the TO-bPNA:RNA hybrid exhibited a robust relative quantum yield of 43% (Fig. 4B). This TO-bPNA:URIL hybrid quantum yield exceeds that observed with the RNA aptamer Mango bound to TO-biotin (quantum yield = 14%)^10^. An RNA hairpin construct with a fully base-paired duplex stem (RNAII) does not improve the brightness of the TO-bPNA derivatives (Fig. 4C). While thiazole orange fluorescence turn-on upon RNA binding is expected for non-specific intercalation, this only occurs at higher concentrations^64^; the null response with non-URIL RNA (RNAII) indicates the critical role of bPNA triplex hybridization in fluorogenic binding. All bPNAs gave a strong fluorogenic response to URIL RNAs, but there appeared to be significant differences among bPNA variants^51,58^. Further, despite the biophysical advantage previously observed for triplex hybridization with L,D and D,L dipeptide bPNAs^51^, these fluorogenic variants were not as bright as the tripeptides (K^2M^AlaK^2M^, K^2M^IleK^2M^). Based on these results, we chose the TO-K^2M^AlaK^2M^ probe (**1**) for subsequent intracellular studies.

### Intracellular labeling of URIL RNAs and bacteriophage RNPs with fluorogenic bPNA

**Fig. 5:**
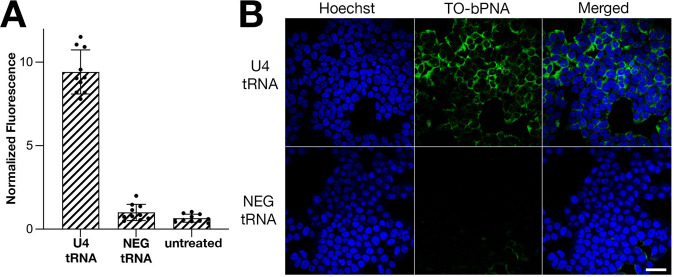
URIL-specific intracellular fluorescence. **A** Integrated fluorescence of U4 tRNA, NEG tRNA and untreated cells normalized to untreated cells. The mean value of 10 independent cell measurements of normalized fluorescence (scatter plot) and standard deviation error is shown. All cell samples were treated with TO-bPNA **1**. **B** Representative confocal fluorescence microscopy images from triplicate studies of HEK-293T cells treated with **1** in media and transfection with plasmid encoding (Top row) U4 tRNA and (Bottom row) NEG tRNA. 170/177 (97%) of cells were labeled with TO-bPNA. Scale bar = 50 µm.

Using similar design principles as the in vitro studies, plasmid vectors were constructed to encode tRNA^Lys ^with URIL RNA hairpins (Fig. 3B, Supplementary Fig. 1) installed in place of the anticodon loop; this platform affords stable intracellular RNA expression^7^ upon transfection into HEK-293T cells. A construct encoding a tRNA scaffold with a fully base-paired stem in place of the URIL (NEG tRNA) was designed to serve as an intracellular negative control that lacks bPNA binding. The U4-tRNA construct was used to determine intracellular fluorogenic triplex hybridization with TO-bPNA. Indeed, treatment of HEK-293T cells with probe **1** in cell culture media following transfection with either U4 or NEG tRNA resulted in a 5- to 10-fold brighter fluorescence intensity in the U4-tRNA transfected cells (Fig. 5, Supplementary Figs. 9 and 10). These data were supportive of intracellular targeting of URIL RNA, albeit with diminished enhancement relative to in vitro conditions.

**Fig. 6:**
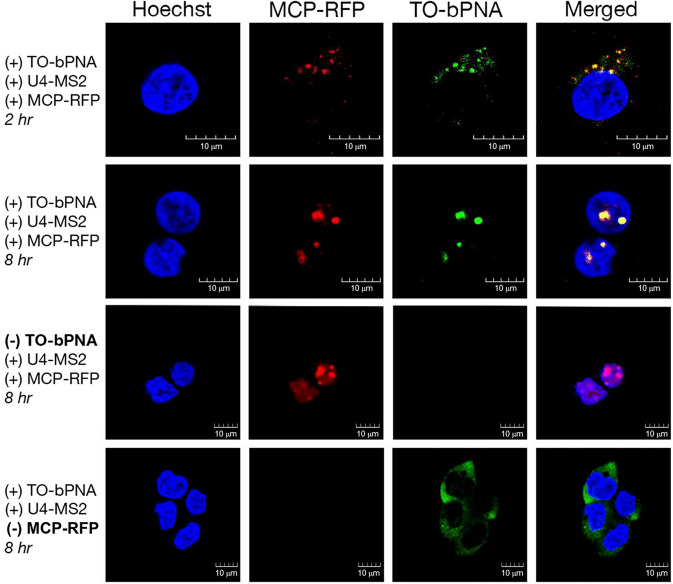
Simultaneous MS2 and FLURIL imaging of RNPs using MCP-RFP and TO-bPNA. Representative confocal fluorescence microscopy images of HEK-293T cells treated as indicated at the left of each row and imaged under the dye channels indicated at the top of each column. Experiments were performed in triplicate. Scale bar = 10 µm. The MCP-RFP fusion contains a nuclear localization tag. The first row is imaged 2 h after transfection and treatment with TO-K^2M^Ala-K^2M^ (1 µM) in media, while subsequent rows are imaged 8 h after transfection.

To benchmark bPNA labeling of RNA against known RNA tracking strategies, we juxtaposed the U4 URIL with the MS2 hairpin sequence in the tRNA^Lys^ scaffold to yield a construct encoding U4-MS2 tRNA (Fig. 3B, Supplementary Fig. 1). The U4-MS2 tRNA plasmid was co-transfected into HEK-293T cells with a plasmid encoding an MCP-RFP fusion, which also bears a nuclear localization signal (NLS)^65^. Cells imaged 2 h after transfection and treatment in media with **1** revealed clear co-localization of red and green fluorescence, supportive of labeling of the U4-MS2 tRNA with TO-bPNA and subsequent binding of the MCP-RFP protein fusion to the triplex hybrid in the cytoplasm (Fig. 6, Supplementary Figs. 5, 6 and 11). This red-green co-localization was accompanied by a condensation of signal intensity, possibly due to RFP aggregation^66^. Notably, imaging 8 h after transfection indicated that both green and red fluorescence had co-localized to the nucleus, consistent with time-dependent transport of both MCP-RFP (which bears the NLS) along with the TO-bPNA hybrid with U4-MS2 tRNA. Thus, treatment of cell culture with TO-bPNA in media can effectively FLURIL-tag intracellular RNA and RNP targets and track RNP transport from cytoplasm to nucleus. Importantly, without MCP-RFP, green fluorescence from TO-bPNA remains cytoplasmic; without TO-bPNA, MCP-RFP remains localized to the nucleus as expected.

### Intracellular labeling of mammalian RNPs with fluorogenic bPNA

**Fig. 7:**
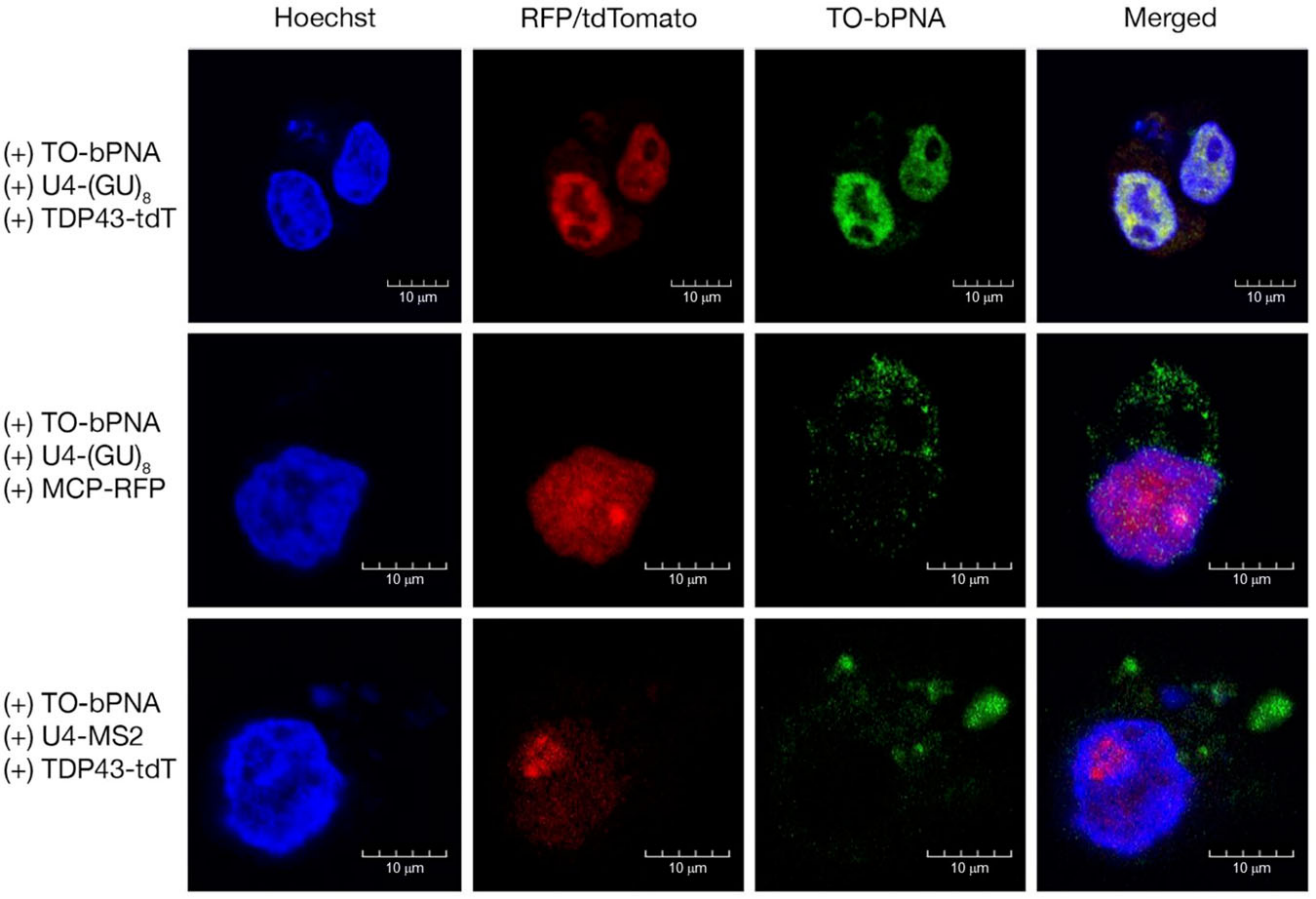
Imaging native RNP pairs using TDP-43-tdTomato and TO-bPNA. Representative confocal fluorescence microscopy images of HEK-293 cells treated as indicated at the left of each row and imaged under the dye channels indicated at the top of each column. Experiments were performed in triplicate. Scale bar = 10 µm. The top row indicates nuclear co-localization of TDP-43-tdTomato with TO-K^2M^Ala-K^2M^ fluorescence when co-expressed with U4-(GU)_8_. Lower two rows feature mismatches between RNA and RBP (U4-(GU)_8_ with MCP-RFP and U4-MS2 with TDP-43-tdTomato, respectively), resulting in partitioning of TO-K^2M^Ala-K^2M^ emission to the cytoplasm.

To complement the bacteriophage RBP system, we conducted an identical experiment with a native mammalian RNA and protein pair to verify intracellular targeting by TO-bPNA. There have been extensive studies on TAR DNA/RNA binding protein (TDP-43) due to its central role in the progression of neurodegenerative diseases such as ALS^67–69^, where it is mislocalized from the nucleus to the cytoplasm^70^. As TDP-43 is known to bind to repeat sequences of UG/TG^71,72^, we co-transfected HEK-293T cells with plasmids encoding URIL-UG repeat RNA (U4-(GU)_8_, Fig. 3B, Supplementary Fig. 1) and TDP-43-tdTomato^73^. Upon Treatment with TO-bPNA in cell media resulted in co-localized red (tdTomato) and green fluorescence in the nucleus (Fig. 7, Supplementary Fig. 8). This is supportive of successful FLURIL tagging of the TDP-43/(GU)_8_ mammalian RNP, in the correct subcellular compartment. With the MS2/MCP and (GU)_8_/TDP-43 systems verified, we transfected with swapped RNA-protein pairings that should not result in complex formation: U4-MS2 was co-transfected with TDP-43-tdTomato, and U4-(GU)_8_ with MCP-RFP (Fig. 7). In these control experiments, both red fluorescent proteins were retained in the nucleus, but the TO-bPNA tagged RNAs remained cytoplasmic, indicating that RNA-protein binding is driving co-localization of red and green fluorescence signals.

### Live cell tracking of genomic loci with bPNA-labeled CRISPR-dCas/gRNA complexes

**Fig. 8:**
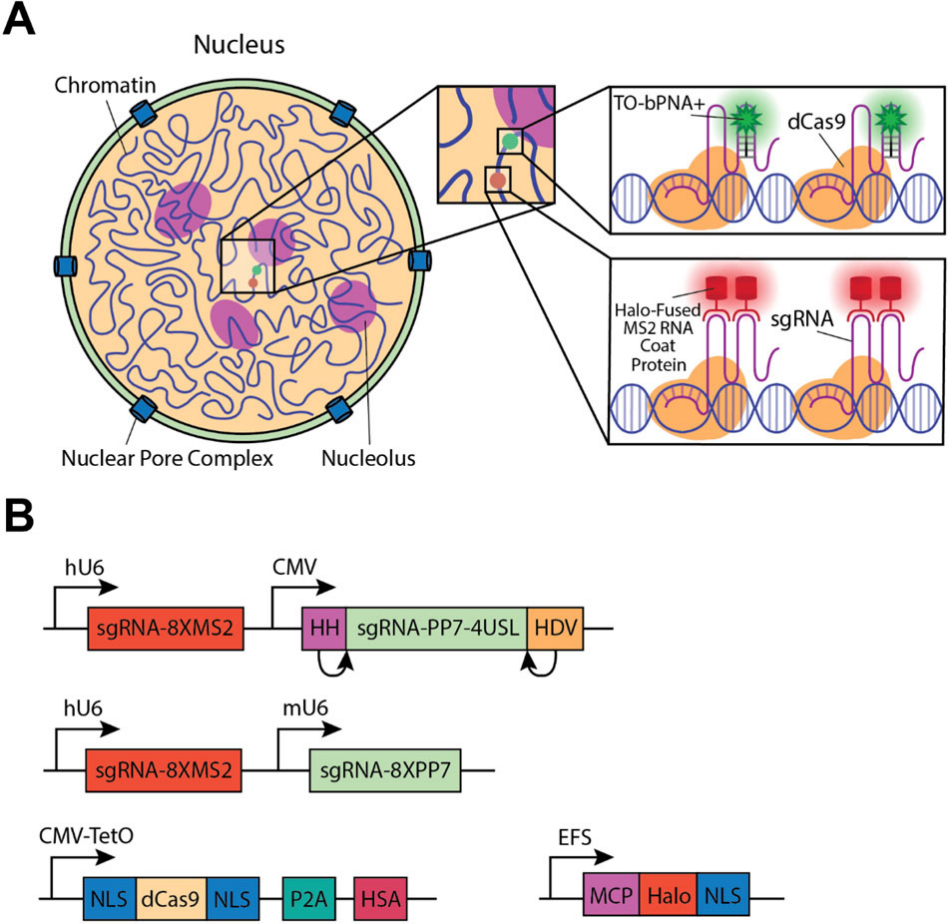
FLURIL tags in CRISPR-dCas live cell genomic loci tracking. **A** Illustration of dual-color genomic labeling of IDR2 and IDR3 by CRISPR-dCas9 targeting. IDR3 is tracked by FLURIL tagging of IDR3-targeting gRNA, while MS2 labeling of IDR2-targeting gRNA is used to track IDR2. FLURIL tags are stained with TO-bPNA, while MS2 labels are stained with MCP-HaloTag binding and HaloTag-JF549 reaction with the complex. MS2 labeling is accomplished using the CRISPR-Sirius gRNA design in Sirius-IDR2-(MS2)_8_ gRNA, which has an array of eight MS2 hairpins. **B** Plasmids used: (Top) dual gRNA plasmid driven by two promoters (hU6 for Sirius-IDR2-(MS2)_8_, CMV for IDR3-U4), (Middle) control dual gRNA plasmid with IDR3-U4 gRNA replaced by Sirius-IDR3-(PP7)_8_, (Bottom) plasmids carrying dCas9 under an inducible promoter, MCP-Halotag fusion under continuous expression. NLS nuclear localization signal, P2A cleavage peptide, HSA mouse heat-stable antigen.

To test the scope of bPNA cell imaging, we incorporated TO-bPNA reporting into an established CRISPR imaging approach for live cell genomic loci tracking^30^. CRISPRainbow is a live cell imaging strategy that uses MS2 or PP7 modified guide RNA (gRNA) with deactivated Cas9 (dCas9) to precisely track endogenous DNA repeats marking specific genomic loci^74^. Incorporation of MS2/PP7 bacteriophage RNA hairpin sequences into the gRNA enables the dCas-gRNA complexes to be tracked in live cells by fluorescence imaging upon co-expression of phage coat protein (MCP/PCP) fusions with a “rainbow” of fluorescent proteins; sequence-optimization of the bacteriophage hairpin array yielded more stable and brighter imaging systems known as CRISPR-Sirius gRNAs^75^. We selected two CRISPRainbow/Sirius gRNAs that target proximal intergenic DNA regions (IDR2, IDR3) and modified one to utilize FLURIL tagging only, with the other serving as an internal benchmark of MS2 labeling. On the gRNA targeting IDR3, a single U_4_XU_4_ bulge (URIL) was installed in a stabilized hairpin (Fig. 3C, Supplementary Fig. 2), rendering the construct (IDR3-U4) trackable by FLURIL tagging. The IDR3-U4 gRNA carries a PP7 hairpin that serves as an internal control for labeling experiments that lack a bPNA probe and was indeed found to be unreactive to labeling in the absence of TO-bPNA. To target IDR2, a CRISPR-Sirius gRNA called Sirius-IDR2-(MS2)_8_ was used (Fig. 3D), which features an array of eight MS2 hairpins optimized for stability. A plasmid was constructed encoding both IDR3-U4 and Sirius-IDR2-(MS2)_8_, and this vector was transfected into U2OS cells stably expressing dCas9 and MCP-HaloTag. Staining of live cells in culture with TO-bPNA and HaloTag-JF549 dye thus enabled simultaneous and orthogonal imaging of IDR3 and IDR2, respectively (Fig. 8A).

**Fig. 9:**
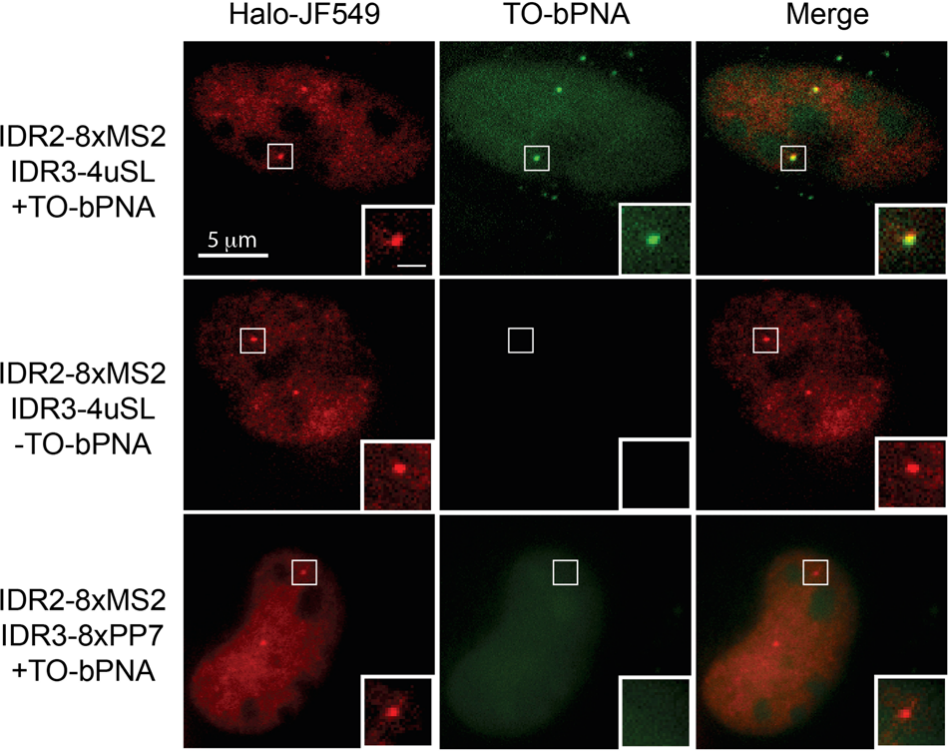
Dual-color CRISPR imaging of IDR2 and IDR3 genomic loci in U2OS by simultaneous FLURIL tagging and MS2 labeling of gRNAs. (Top row) Representative loci imaging from triplicate independent measurements, following transfection with a dual plasmid encoding Sirius-IDR2-(MS2)_8_ and IDR3-U4, followed by staining with Halo-JF549 (red) and TO-bPNA (green), respectively. Genomic loci highlighted in the white square are shown enlarged, inset bottom right-hand corner. (Middle row) Same plasmid transfection as top row without TO-bPNA treatment. (Bottom row) Loci imaging following transfection with a dual plasmid encoding Sirius-IDR2-(MS2)_8_ and Sirius-IDR3-(PP7)_8_, followed by staining with Halo-JF549 (red) and TO-bPNA (green), respectively. Bar in the inset, 1 μm.

**Fig. 10:**
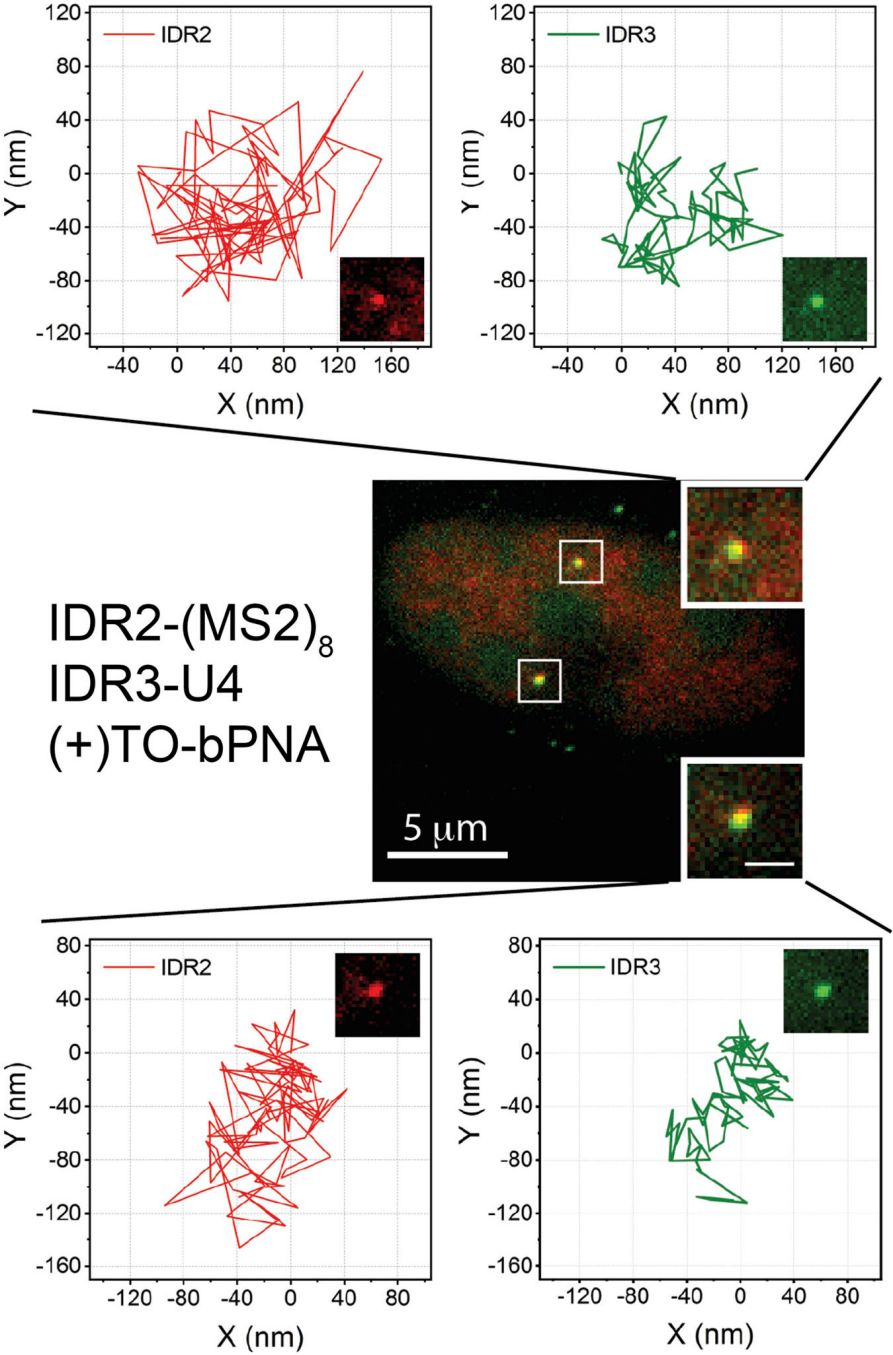
FLURIL-tag tracking of IDR2 and IDR3 dynamics in live cells. U2OS cells were imaged 48 h post-transfection with a dual plasmid encoding Sirius-IDR2-(MS2)_8_ and IDR3-U4 and stained with HaloTag-JF549 (red) and TO-bPNA (green). Representative loci trajectories of 96 frames from homologous chromosomes are shown in the top and bottom from triplicate independent measurements. The imaging rate is 136 ms per frame. Bar in the inset, 1 μm. For comparison, trajectories were aligned to start from the origin (0,0).

The final vector design was driven by the low efficiency of initial efforts in the expression of the modified gRNAs. We speculated that the oligo-U domains triggered early termination by RNA Pol III, which is known to significantly reduce the gRNA targeting efficiency in the CRISPR system^65,76^. To bypass this issue, we expressed gRNA under a CMV Pol II promoter and ensured precise transcript generation by flanking the gRNA sequence with Hammerhead (HH) and Hepatitis Delta Virus (HDV) self-cleaving ribozymes^77^. We thus modified the gRNA plasmid to contain the Hammerhead (HH) and Hepatitis delta virus (HDV) ribozymes on either side of the gRNA under a CMV promoter and inserted it into a dual gRNA plasmid expressing CRISPR-Sirius gRNA (Sirius-IDR2-(MS)_8_) for dual-color genomic loci targeting (Fig. 8). Indeed, the resulting dual-color genomic loci labeling system for IDR2 (Sirius-IDR2-(MS2)_8_ + MCP-Halotag + Halotag-JF549) and IDR3 (IDR3-U4 + TO-bPNA) was readily established in U2OS cells by lipofectamine plasmid transfection and staining with dyes delivered in media (HaloTag-JF549, TO-bPNA). Live cell imaging revealed partially overlapped red (IDR2, HaloTag-JF549) and green (IDR3, TO-bPNA) foci (Fig. 9, top row, Supplementary Fig. 4), which was expected given the close proximity (~4.6 kB) of the two loci on chromosome 19. The exclusion of TO-bPNA from media resulted in the loss of IDR3 foci, leaving only red IDR2 foci visible (Fig. 9, middle row). Additionally, identical plasmid transfection protocols were carried out in which the FLURIL gRNA was replaced with a CRISPRainbow gRNA bearing PP7 hairpins (Sirius-IDR3-8xPP7). When stained with HaloTag-JF549 and TO-bPNA, only red foci were visible (Fig. 8, bottom row), consistent with URIL-selective fluorogenic binding of the TO-bPNA probe previously observed in fixed cells (Fig. 5). FLURIL-tagged IDR3 loci was easily tracked over 13 s without significant photobleaching or dye blinking, performing comparably to IDR2 loci tracking with the established CRISPR-Sirius (8X-MS2) system^75^. Time-lapse imaging was carried out for 96 frames at a capture rate of 136 ms per frame (Supplementary Movie S1), yielding IDR2 and IDR3 trajectories on identical and homologous chromosomes of similar range (~100–200 nm) but in different patterns of loci territories, consistent with the labeling of proximal, but distinct loci (Fig. 10). FLURIL tagging of gRNA thus gave a highly usable fluorescence signal-to-noise ratio with single locus labeling, likely due to minimal background fluorescence from unbound TO-bPNA, unlike constitutively fluorescent FP and HaloTag systems. Though single molecule fluorescence tracking has not been demonstrated, we note that the sole FLURIL tag was sufficient for labeling the low-copy number IDR3 locus (45 copies over 1.5 kB). Under standard operating conditions, the low background of a single FLURIL gRNA tag affords 7X greater brightness (Supplementary Fig. 3) than the CRISPR-Sirius gRNA tags that have a substantially larger molecular footprint of eight bacteriophage RNA hairpins bound by bacteriophage coat protein fusions.

## Discussion

Taken together, these data establish FLURIL tagging as an efficient and convenient strategy for intracellular RNA, RNP, and DNA tracking that leverages selective URIL targeting with fluorogenic binding to establish a robust and bright RNA tracking signal. We find that TO-bPNA exhibits significant (~600X) enhancement of emission intensity upon bPNA triplex hybridization with structured RNAs URILs in vitro and a substantive increase in quantum yield from ~0.01% in the unbound state to 43% in the RNA complex. This significant fluorogenic response to URIL-RNAs translated to 5–10X intracellular signal-to-noise and a highly usable, low-background cellular imaging of URILs. While an engineered biomolecule (URIL-RNA) is needed, this is a universal requirement for the most broadly used, genetically encoded live cell tracking strategies such as MS2-labeling.

Intracellular FLURIL tagging of RNAs in both fixed and live cells was directly verified using established protein labels from endogenous mammalian RNPs as well as bacteriophage MS2 labeling, which remains the gold standard for RNA tracking. In the present study, we have inserted stabilized hairpin structures to host the URIL site and included a PP7 hairpin as a control element in IDR3-U4; however, a URIL could be inserted into a native fold by replacement of an existing native 4 bp stem buttressed by other structures in the ROI, further streamlining the FLURIL-tag footprint. Importantly, RBP fusions with fluorescent proteins (FP) confirmed that FLURIL tags were labeling the correct RNA species, while cells lacking the correct RNA-protein pairing resulted in the segregation of FLURIL tag and RBP-FP fusion signals. Under live cell tracking conditions, FLURIL tags also performed comparably to established MS2-based methods (CRISPR-Sirius) while occupying a considerably more compact molecular footprint. It is particularly noteworthy that the FLURIL tag only requires the replacement of a 4 bp stem with an 8-nt URIL to enable RNP live cell tracking with a cell-permeable, ~1kD bPNA probe.

Though there remain many appealing aspects to MS2 labeling, including multiplex and multicolor approaches, the use of protein fusions and multiple RNA hairpin sites encumbers the RNA of interest with substantial steric bulk, adding 41–47 kD of mass per MCP-FP and MCP-HaloTag fusion, respectively. The steric bulk and RNA secondary structures required for protein labeling have raised concerns that this could affect native transcript processing and tertiary contacts^1^; however, inhibition of transcript degradation by insertion of bacteriophage (MS2/PP7) hairpins is less of an issue in mammalian cells^22,27,28^. In contrast, FLURIL tags add negligible additional mass to the RNA target and a structural perturbation that may be as minor as the replacement of a duplex stem with a triplex stem. Prior work has demonstrated that this replacement does not disrupt proximal RNA domains, retaining tertiary interactions as well as catalytic function. In addition to minimizing the addition of obtrusive non-native structures, FLURIL tagging also avoids the use of labile^21^ G-quadruplex domains found in SELEX-derived dye-binding aptamers such as Spinach and Mango. Furthermore, as fluorogenic dye binding is driven by bPNA triplex hybridization to the URIL rather than direct RNA recognition of the dye, prosthetic groups^50^ may be used to decorate RNPs without the technical demand of a SELEX campaign. In particular, other fluorogens in the cyanine family could potentially be used in FLURIL tagging, thus expanding the color range available without laborious aptamer selection procedures. Analogously, the aptamer Pepper^11^ binds a family of fluorogenic dyes with significant structural variation, suggesting that minimal specific contacts are needed to access fluorogenic binding.

The unique advantages of FLURIL tagging are counterbalanced by some limitations. As mentioned above, a URIL motif must be installed in the RNA of interest, requiring non-native RNA expression by transfection. The efficiency of tracking, as described herein, thus depends on the efficiency of transfection and vector design, which can lead to variability in labeling outcomes; additionally, transfection yields elevated, non-native transcript levels. These issues may be alleviated with genome editing, though this comes with a significant cost and effort per experiment. Currently, FLURIL tagging has only been demonstrated with a single color, though efforts to expand both color selection and sequence scope^41^ are underway. Despite these caveats, the present work demonstrates the utility of FLURIL tagging, as well as its appealing compatibility with popular methods such as MS2/Halotag labeling. We anticipate that FLURIL tagging will be a useful tool that may also be used in conjunction with other methods (eg-dye aptamers, Riboglow, dCas13, FISH) to facilitate RNA tracking while minimizing the steric burdens of RNA labeling.

## Methods

### Materials and handling

All chemicals were used without further purification from commercial sources as indicated unless otherwise noted. DNAs and RNAs were purchased from Integrated DNA Technologies (IDT). Nucleic acid strands shorter than 20 nt were used without further purification. Otherwise, the DNAs/RNAs were purified by TBE-urea denaturing gel. DNA stock solutions were serially diluted in MilliQ water, and concentrations were determined by measuring solution absorbance at 260 nm on a Thermo Fisher Nanodrop 2000. Sample fluorescence was measured on a Thermo Fisher Nanodrop 3300. Cell lines were acquired from ATCC (HEK-293T, cat# CRL-2316; U2OS, cat# HTB-96) and cultured according to ATCC protocols.

### Nucleic acid sequences

RNAs for in vitro fluorescence studies shown below were purchased. All RNAs were annealed prior to use. Duplexes were annealed together from A and B strands indicated.

12-U4-12 A: 5’-CGCAUAGCUCAGUUUUGACUCGAUACGC-3’

12-U4-12 B: 5’-GCGUAUCGAGUCUUUUCUGAGCUAUGCG-3’

12-U6-12 A: 5’-CGCAUAGCUCAGUUUUUUGACUCGAUACGC-3’

12-U6-12 B: 5’-GCGUAUCGAGUCUUUUUUCUGAGCUAUGCG-3’

RNAI U6: 5’-GGCAGCUUUUUUUUGGUAGUUUUUUGCUGCC-3’

RNAII WT: 5’-GCACCGCUACCAACGGUGC-3’

The following RNA sequences were delivered into cells by plasmid transfection for intracellular labeling.

U4 tRNA: 5’-GCCCGGAUAGCUCAGUCGGUAGAGCAGCGGCCGUUUUCGCUCCGGCGUUUUCGGCCGCGGGUCCAGGGUUCAAGUCCCUGUUCGGGCGCCA-3’

U4-MS2 (MBSV5) tRNA: 5’-GCCCGGAUAGCUCAGUCGGUAGAGCAGCGGCCGUUUUCGCACAUGAGGAUCACCCAUGUGCGUUUUCGGCCGCGGGUCCAGGGUUCAAGUCCCUGUUCGGGCGCCA-3’

NEG tRNA: 5’-GCCCGGAUAGCUCAGUCGGUAGAGCAGCGGCCGCGCGCGCUCCGGCGCGCGCGGCCGCGGGUCCAGGGUUCAAGUCCCUGUUCGGGCGCCA

(GU)_8_-U4 RNA: 5’-CGGCCGUUUUCGCUCCGGCGUUUUCGGCCGGUGUGUGUGUGUGUGU-3’

### In vitro fluorescence measurement

RNAs were annealed with bPNA at a 1:1 ratio and incubated for 30 min following slow cooling (50 mM HEPES, pH 7.5, 100 mM NaCl, 2 μM RNA, 2 μM bPNA). Sample fluorescence was measured using a Thermo Fisher Nanodrop 3300 (relative fluorescence units (RFU), excitation = 470 nm, emission = 522 nm, 2 μl sample volume). All fluorescence values are the average of triplicate measurements, starting from fresh sample preparation. Error bars indicate standard deviation. Data were processed using OriginPro and Graphpad Prism.

### Quantum yield measurement

Relative quantum yield was calculated against fluorescein (quantum yield = 92%)^78^. All dye samples (TO, TO-bPNA) were prepared at 1 μM with hybrid samples prepared at 1:1 TO:RNA ratio (50 mM HEPES, pH 7.5, 100 mM NaCl). Sample concentrations were determined by UV absorbance (Cary UV-Vis-NIR Spectrophotometer) and adjusted to obtain matched sample absorbances, not to exceed 0.04 at 470 nm (±0.0002). The fluorescence was measured by Quantamaster 8000 spectrofluorometer and corrected for anisotropy with a vertical polarizer in the excitation path (470 nm) and a polarizer in the emission path (507 nm) set at the magic angle (54.7°), with excitation and emission slit widths = 5 nm)^79^. The integrated emission of the bPNA hybrid was 49.7% that of fluorescein, indicating a relative quantum yield of 43%.

### Plasmid construction

The DNA inserts corresponding to modified tRNA scaffolds (U4, U4-MS2, NEG tRNA) were annealed into duplexes from which 2 µg were digested with SalI and XbaI (Thermo Fisher, 2x Tango buffer, 12 h), following Thermo Fisher protocol. Vector pAV U6 + 27 (1 µg) was separately digested with SalI and XbaI (12 h), and all digested products were gel purified (band isolation from with Qiagen QIAquick Gel Extraction Kit). Purified products were ligated (DNA insert:linearized vector=5:1) with T4 DNA ligase (Thermo Fisher), following published protocol^80^. The ligation product was transformed into DH5α for amplification, and the resulting mixture was inoculated on an agar plate with ampicillin selection. After 16 h, several colonies were picked and amplified in LB media containing ampicillin. The harvested plasmid was isolated by miniprep (Qiagen) and verified by sequencing. Tomato-TDP43 and MCP-TagRFPt plasmids were obtained from Addgene (#28205 and #64541)^73,81^ and amplified in the corresponding E.coli cell lines.

### Cell treatment (fixed cell analysis)

HEK-293T cells were cultured based on ATCC protocol. Cells were seeded to a 35-mm culture dish (Thermo Fisher) with a clean coverslip (Thermo Fisher) attached to the bottom at a concentration of 5 × 10^5^/ml. After one day of incubation, the cells were transfected with corresponding plasmids (1000 ng each per dish) with Lipofectamine 3000 (Invitrogen) following the online protocol, and the cells were incubated for 24 h. Culture medium was removed and the fresh medium containing 1 µM bPNA-dye was added to the attached cells. The cells were incubated at 37 °C for 2–8 h, medium discarded, and Hoechst 33258 (Invitrogen) in fresh culture medium was added at the final concentration of 200 ng/ml to the cells and incubated for 15 min at 37 °C. Cells were washed with PBS, fixed with 4% formaldehyde in PBS for 15 min at room temperature and rinsed with PBS. The coverslip was transferred to glass slides for fluorescence microscopy and imaged under Olympus FV3000 systems (Objective lens: ×40, Zoom: ×1. Blue channel: (excitation) 405 nm, (emission) 422 nm, Voltage: 355 V, detection range: 420–470 nm; Green: (excitation) 488 nm, (emission) 520 nm, Voltage: 580 V, detection range: 499–599 nm). Three or more replicate data sets were obtained that showed consistent findings, starting from cell seeding.

### Fluorescence microscopy (live cell imaging)

Cell imaging (U2OS) was carried out on an Olympus IX83 microscope equipped with three EMCCD cameras (Andor iXon 897) mounted on a 4-camera splitter, four lasers (405 nm, 488 nm, 561 nm and 647 nm), mounted with a ×1.6 magnification adapter and ×60 apochromatic oil objective lens (NA 1.5), resulting in a total of ×96 magnification. The microscope stage incubation chamber was maintained at 37 °C with CO_2_ and humidity supplement. A laser quad-band filter set for TIRF (emission filters at 445/58, 525/50, 595/44, 706/95) was used to collect fluorescence signals simultaneously. Data acquisition was carried out with CellSens 4.1.1 software. Localization precision was ~5 nm in 4 s, ~6 nm in 16 s, and ~10 nm in 80 s^82^. The video was recorded at 136 ms per frame with a total of 96 frames and 100 ms exposure time. Image size was adjusted to show individual nuclei, and intensity thresholds were set on the basis of the ratios between nuclear foci signals to background nucleoplasmic fluorescence.

### Image processing

The images were registered and analyzed by *Fiji*^83^ and *Mathematica* (Wolfram) software. To achieve subpixel registration accuracy, parameters for shifting, scaling, and rotating camera images were determined by the least-squares fitting of fluorescent bead images (100 nm TetraSpeck fluorescent microspheres, Invitrogen). The experimental data from each channel were processed through an affine transformation and overlapped in false-color channels for visualization. The locus trajectory was obtained by tracking the locus position over time, and graphs were generated by *OriginPro* (OriginLab version 2019b).

### Reporting summary

Further information on research design is available in the Nature Portfolio Reporting Summary linked to this article.

## Supplementary information

Supplementary Information

Reporting Summary

Supplementary Movie S1

## Data Availability

All data that support this manuscript, including source data files, are available in the Supplementary Information. Source data are provided with this paper.

## Source data

Source data
