# Supplementary material for: Intracellular RNA and DNA tracking by uridine-rich internal loop tagging with fluorogenic bPNA
Source: Nat Commun. 2023 May 24;14:2987. doi: 10.1038/s41467-023-38579-2 (PMC10209184; doi:10.1038/s41467-023-38579-2)
Supplement: Supplementary file 1 — Supplementary Information [file 41467_2023_38579_MOESM1_ESM.pdf]

SUPPLEMENTARY INFORMATION

Intracellular RNA and DNA tracking by uridine-rich internal loop tagging with fluorogenic bPNA

Yufeng Liang,[1,2] Sydney Willey,[2,3,4] Yu-Chieh Chung,[2,3,4] Yi-Meng Lo,[1,2] Shiqin Miao,[1,2] Sarah Rundell,[1,2] Li-Chun Tu[2,3,4]* and Dennis Bong[1,2] *

[1]Department of Chemistry & Biochemistry, The Ohio State University, Columbus, OH, USA
[2]Center for RNA Biology, The Ohio State University, Columbus, OH, USA
[3]Department of Biological Chemistry and Pharmacology, The Ohio State University, Columbus, OH, USA
[4]The Ohio State University Comprehensive Cancer Center, The Ohio State University, Columbus, OH, USA

*Correspondence:
tu.277@osu.edu
bong.6@osu.edu

Table of contents

Section		Page
**S1**	Materials and handling	2
**S2**	General experimental procedures	4
**S3**	Additional fluorescence microscopy data (fixed cells)	7
**S4**	Additional in vitro fluorogenic binding data	9
**S5**	Synthetic procedures	10
**S6**	Compound characterization	14

S1. General

All chemicals were used without further purification from commercial sources as indicated, unless otherwise noted. DNAs and RNAs were purchased from Integrated DNA Technologies (IDT). Nucleic acid strands shorter than 20 nt were used without further purification. Otherwise, the DNAs/RNAs were purified by TBE-urea denaturing gel. SYBR® gold was purchased from Thermo Fisher Scientific. DNA stock solutions were serially diluted in MilliQ water and concentrations were determined by measuring solution absorbance at 260 nm on a Thermo Fisher Nanodrop 2000. Sample fluorescence was measured on a Thermo Fisher Nanodrop 3300. Microscopy images are shown in green and red color to correspond with green and red emission wavelengths as in green fluorescent protein (GFP), red fluorescent proteins (RFP, dTomato).

S1.1. Nucleic acid sequences

- RNAs for *in Vitro* fluorescence turn-on

A and B strands were annealed together into duplexes before use.

12-U4-12 A: 5’-CGCAUAGCUCAGUUUUGACUCGAUACGC-3’
12-U4-12 B: 5’-GCGUAUCGAGUCUUUUCUGAGCUAUGCG-3’

12-U6-12 A: 5’-CGCAUAGCUCAGUUUUUUGACUCGAUACGC-3’
12-U6-12 B: 5’-GCGUAUCGAGUCUUUUUUCUGAGCUAUGCG-3’

RNAI U6: 5’-GGCAGCUUUUUUUUGGUAGUUUUUUGCUGCC-3’

RNAII WT: 5’-GCACCGCUACCAACGGUGC-3’

- Plasmid delivered RNA sequences for intracellular labeling

U4 tRNA:
5’-GCCCGGAUAGCUCAGUCGGUAGAGCAGCGGCCGUUUUCGCUCCGGCGUUUUCGGCCGCGGGUCCAGGGUUCAAGUCCCUGUUCGGGCGCCA-3’

U4-MS2 (MBSV5) tRNA:
5’-GCCCGGAUAGCUCAGUCGGUAGAGCAGCGGCCGUUUUCGCACAUGAGGAUCACCCAUGUGCGUUUUCGGCCGCGGGUCCAGGGUUCAAGUCCCUGUUCGGGCGCCA-3’

Negative tRNA:
5’-GCCCGGAUAGCUCAGUCGGUAGAGCAGCGGCCGCGCGCGCUCCGGCGCGCGCGGCCGCGGGUCCAGGGUUCAAGUCCCUGUUCGGGCGCCA

$(GU)_8$-U4 RNA: 5’-CGGCCGUUUUCGCUCCGGCGUUUUCGGCCGGUGUGUGUGUGUGUGU-3’

Predicted secondary structures based on previously reported parameters and using ViennaRNA package are shown below in Table S1.[1–3]

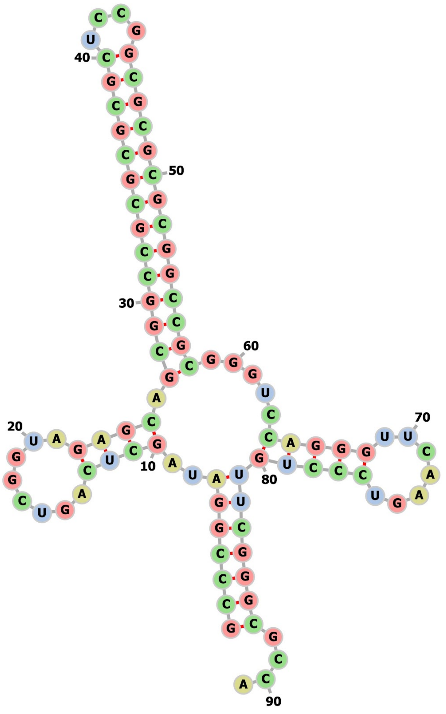

NEG tRNA predicted fold.

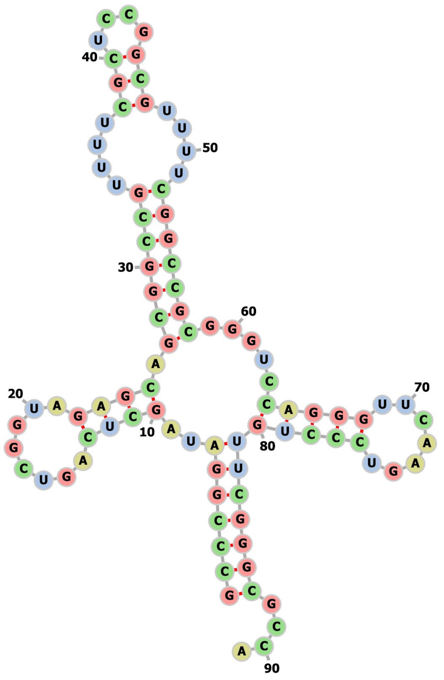

U4-tRNA predicted fold.

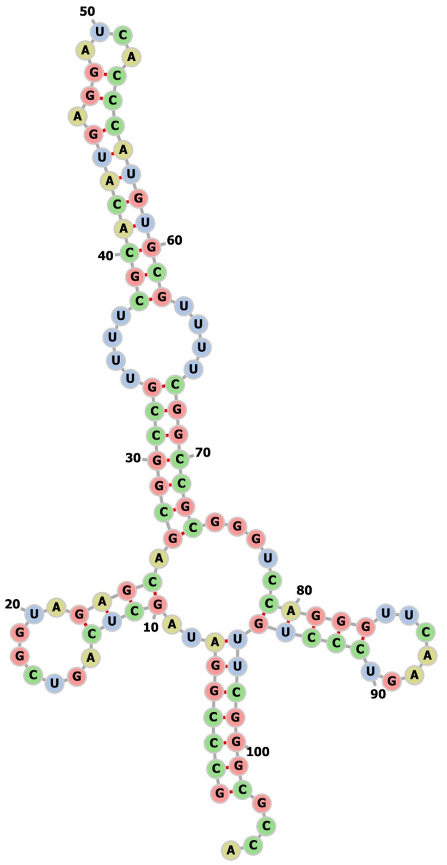

MS2-U4 tRNA predicted fold.

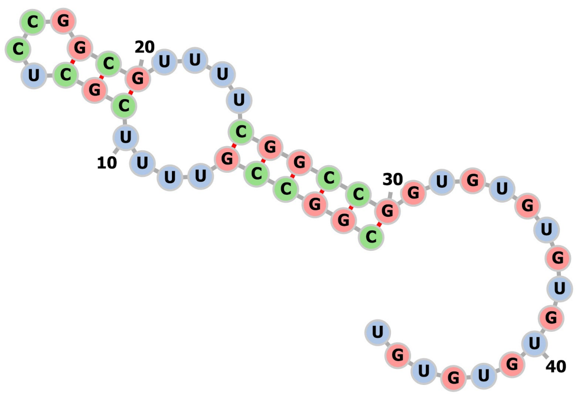

U4-(GU)$_8$ predicted fold.

Supplementary Figure 1. Predicted RNA secondary structures for URIL RNA probe constructs.[1–3]

S2. General experimental procedures

S2.1. HPLC

Fluorogenic bPNA probe was purified using HPLC: Hitachi D-7000 (interface), Hitachi L-7150 (UV-detector) and Hitachi L-7400 (pump). Analytical and semi-preparative HPLC was carried out with C_{18} reverse phase columns. HPLC solvent A: 99% MilliQ H_2O, 1% HPLC grade acetonitrile, 0.1% trifluoroacetic acid; HPLC solvent B: 10% MilliQ H_2O, 90% HPLC grade acetonitrile, 0.07% trifluoroacetic acid.

S2.2. In vitro fluorescence turn-on measurement

Samples were prepared as follows: 50 mM HEPES (pH 7.5), 100 mM NaCl, 2 µM RNA, 2 µM probe. The nucleic acids were annealed from 95 °C to pre-form the structure before use. The samples were prepared and incubated at room temperature for 30 min before measuring the fluorescence (RFU) with Thermo Fisher Nanodrop 3300 (excitation=470 nm, emission=522 nm). For each measurement, 2 µL sample volume was applied to the center of the nanodrop probe. All fluorescence values are the average of triplicate measurements, starting from fresh sample preparation. Error bars indicate standard deviation.

S2.3. Fitting methodology for binding curves

Probes (bPNA-TO) were held at a constant concentration of 100 nM and treated with RNA to final concentrations from 0 to 1.4 µM in buffer (50 mM HEPES, pH 7.5, 100 mM NaCl). The experiments were triplicated from sample preparation and error bars indicate standard deviation. The data were fit with the following equation (1) to obtain dissociation constant Kd :

$$RFU = \frac{m*(Kd+x+100)-m*sqrt[sqr(Kd+x+100)-4*x*100]}{2} \qquad (1)$$

S2.4. Quantum yield measurement

Fluorescence measurements were taken to obtain relative quantum yield using fluorescein as a standard, which has similar excitation and emission to thiazole orange and a known quantum yield of 92% under the experimental conditions.[4] All samples were prepared in 50 mM HEPES (pH 7.5), 100 mM NaCl. RNA used was 12-U4-12 duplex (S1.1). Hybrid samples contained 1 µM RNA, 1 µM TO-bPNA probe; fluorogenic TO-bPNA and free thiazole orange without RNA samples were prepared at 1µM concentration as well. The RNA samples were annealed by slow cooling from 95°C to pre-form the structure before use. The samples were prepared and incubated at room temperature for 30 min before measuring the absorbance with Cary Series UV-Vis-NIR Spectrophotometer. The standard fluorescein was prepared in 0.1M NaOH. All sample concentrations were adjusted to obtain matched sample absorbances, not to exceed 0.04 at 470 nm (± 0.0002). The fluorescence was measured by Quantamaster 8000 spectrofluorometer and corrected for anisotropy with a vertical polarizer in the excitation path (470 nm) and a polarizer in the emission path (507 nm) set at the magic angle (54.7°), with excitation and emission slit widths = 5 nm).[5] Emission spectra were collected and integrated, indicating emission from the bPNA hybrid to be 49.7% that of fluorescein and therefore a relative quantum yield of 43%.

S2.5. Construction of RNA plasmid vector

The DNAs were annealed into duplex and from which 2 µg were digested with SalI and XbaI (Thermofisher) in 2x Tango buffer for 12 hours, following protocol provided by Thermofisher. The vector pAV U6+27 (1 µg) was also digested with SalI and XbaI for 12 hours. The digested products were purified on 1% agarose gel and the bands were cut for DNA isolation by QIAquick Gel Extraction Kit (Qiagen). The purified products were ligated (molar ratio of DNA insert:linearized vector=5:1) with T4 DNA ligase (Thermofisher), following the protocol for Corn Aptamer construction.[6] The ligation product was transformed into DH5α for amplification and the transformation mixture was inoculated on Agar plate (Ampicillin). After 16 hours, several colonies were picked and amplified in LB media containing Ampicillin, and the plasmid was isolated by miniprep (Qiagen). The plasmid was sent for sequencing to verify the insertion. Tomato-TDP43 and MCP-TagRFPt plasmids were obtained from Addgene (#28205 and #64541),[7,8] and amplified in the corresponding E.coli cell lines.

S2.6. Cell treatment (fixed cell analysis)

HEK-293 cells were cultured based on ATCC protocol. Cells were seeded to 35 mm culture dish (Thermofisher) with clean coverslip (Thermofisher) attached to the bottom at the concentration of $5x10^5$/ml.

After one day of incubation, the cells were transfected with corresponding plasmids (1000 ng each per dish) with Lipofectamine 3000 (Invitrogen) following the online protocol and the cells were incubated for 24 hr. Then the culture medium was removed and the fresh medium containing 1 μM bPNA-dye was added to the attached cells. The cells were incubated at 37°C for 2-8 hr and the medium was discarded. Hoechst 33258 (Invitrogen) in culture medium was added at the final concentration of 200 ng/ml to the cells and incubated for 15 min at 37°C and the medium was discarded. The cells were washed with PBS, fixed with 4% formaldehyde in PBS for 15 min at room temperature and rinsed with PBS. The coverslip was transferred to glass slides for fluorescence microscopy. Cells were imaged under Olympus FV3000 systems (Objective lens: 40x, Zoom: 1X. Blue channel: (excitation) 405 nm (emission) 422 nm, Voltage: 355 V, detection range: 420 - 470 nm; Green (excitation) 488 nm, (emission) 520 nm, Voltage: 580 V, detection range: 499 - 599 nm). Three or more replicate data sets were obtained that showed consistent findings, starting from cell seeding.

S2.7. Cellular fluorescence quantification (fixed cell)
Using ImageJ software, the fluorescence intensity in a circular area of diameter ~5 μm within a single cell (HEK-293) was selected and the grayscale was reported. For each treatment, 10 areas were selected in this manner from 10 different cells. The intensity from NEG-tRNA plasmid treated cells was normalized to 1.0.

S2.8. Plasmid Construction

The dCas9 (Addgene#121936) and MCP-HaloTag (Addgene#121937) plasmids from prior work in Tu lab were used without modification[9] and expression vector for the guide RNAs (gRNAs) were modified from previous gRNA plasmids. The single gRNA plasmid for IDR3-U4 gRNA was constructed from pLH-sgRNA1-2XPP7[10] (Addgene#75390) where PP7-U4 was inserted to replace 2XPP7 (Figure S1). The dual gRNA plasmid[9] was constructed from pPUR-hU6-Sirius-8XMS2-mU6-Sirius-8XPP7 (Addgene#121944), in which CMV-HH-PP7-4USL-HDV was inserted to replace mU6-Sirius-8XPP7, resulting in pPUR-hU6-Sirius-8XMS2-CMV-HH-PP7-4USL-HDV. The HH and HDV cassettes were inserted for precise gRNA sequences through a cleavage mechanism at a defined position expressed under a pol II promoter in eukaryotic cells. The targeting sequences for IDR2 and IDR3 were 5'-GGAGAGGCTGGG-3' and 5'-AGCAGATGTAGG-3', respectively.[9,11] The gRNA sequences were ordered from IDT and inserted using BbsI restriction enzyme following the same protocol. The modified guide RNA expression vector reported here will be deposited in Addgene.

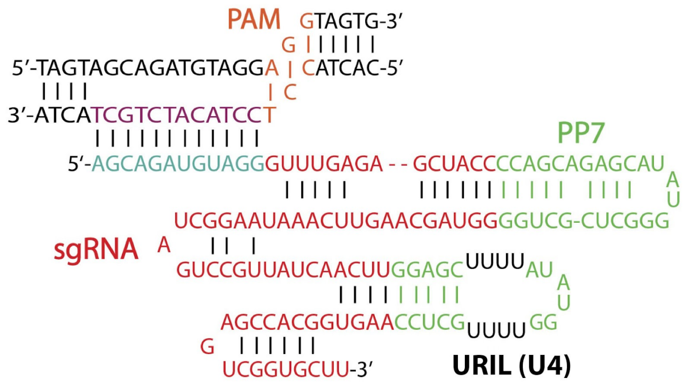

Supplementary Figure 2. Sequence and structure of IDR3-U4 single gRNAs. CRISPRainbow with two PP7 hairpins (2xPP7) gRNA was modified to carry the U_4xU_4 internal bulge (URIL) at the second loop position. The cyan color shows crRNA that targets the endogenous DNA sequences (purple). PAM sequence is shown in orange. Red indicates the gRNA scaffold and PP7 and URIL hairpin scaffolds are shown in green. The PP7 hairpin was carried over from a prior sgRNA construct that was modified and serves as an internal control for labeling experiments that lack bPNA probe.

S2.9. Cell Culture, Lentivirus Production and Transduction (live cell experiments)
We cultured human osteosarcoma U2OS at 37°C in Dulbecco-modified Eagle's Minimum Essential Medium (DMEM) containing high glucose and supplemented with 10% (vol/vol) fetal bovine serum. U2OS$^{dCas9\text{-}HSA/MCP\text{-}HaloTag}$ cell line that was generated by lentiviral transduction and sorted by FACSFusion cell sorter (BD Bioscience, see S2.10 Flow Cytometry). Lentiviral particles carrying dCas9 and MCP-HaloTag were generated by HEK-293 cells using the same protocol.[11] For lentiviral transduction, U2OS cells maintained as described above were transduced by Spinfection in 6-well plates with lentiviral supernatant for 48 hours and ~2x10^5 cells were combined with 1 ml lentiviral supernatant and centrifuged for 30 minutes at 1200 x *g*. For

imaging, U2OS$^{dCas9\text{-}HSA/MCP\text{-}HaloTag}$ cells were grown on 35-mm glass-bottom dishes (MatTek) and 2 µg of sgRNA plasmid were transfected using TransIT transfection reagent (Mirus) following manufacturer's protocol. Cells were washed with fresh media 24 hours post-transfection and imaged after another 24 h incubation. A final concentration of 1 µM TO-bPNA was added to the culture medium two hours before imaging. Cell toxicity was not observed even with prolonged incubation (up to 48 hours) in the presence of TO-bPNA. Cells were tested and found to be free of mycoplasma contamination.

S2.10. Flow cytometry

The cell line U2OS$^{dCas9\text{-}HSA/MCP\text{-}HaloTag}$ was generated using the same protocol[9] that generated U2OS$^{dCas9\text{-}HAS/PCP\text{-}GFP/MCP\text{-}HaloTag}$ with the following modifications: (1) The PCP-GFP for labeling PP7 stem loop was not added; (2) cells expressing the dCas9-p2A-HSA and MCP-HaloTag (stained with HaloTag-JF549) were selected using a BD FACSAria Fusion cell sorter (BD Bioscience) equipped with 405, 488, 561 and 640 nm excitation lasers and standard emission filters for PE (582/15) and APC (670/30); (3) AlexaFluor 647-conjugated anti-mouse CD24 antibody (BioLegend) was used to stain for HSA (heat stable antigen, mouse) carried on the dCas9 plasmid; (4) U2OS$^{dCas9\text{-}HSA/MCP\text{-}HaloTag}$ was not selected from a single cell. FACS sorting for dCas9 positive cells was carried out following sample staining (1 µL Alexa Fluor-647 conjugated anti-mouse CD24 antibody, 100 µL cell solution, 30 min). FACS sorting of MCP-HaloTag positive cells was carried out after staining with HaloTag-JF549 (2 nM dye, 12-24 hr).

S2.11. Fluorescence Microscopy (live cell imaging)

Cell imaging was carried out on an Olympus IX83 microscope equipped with three EMCCD cameras (Andor iXon 897) mounted on a 4-camera splitter, four lasers (405 nm, 488 nm, 561 nm, and 647nm), mounted with a 1.6x magnification adapter and 60x apochromatic oil objective lens (NA 1.5), resulting in a total of 96x magnification. The microscope stage incubation chamber was maintained at 37°C with CO_2 and humidity supplement. A laser quad-band filter set for TIRF (emission filters at 445/58, 525/50, 595/44, 706/95) was used to collect fluorescence signals simultaneously. Data acquisition was carried out with CellSens software. Localization precision was ~5 nm in 4 seconds, ~ 6 nm in 16 seconds, and ~ 10 nm in 80 seconds.[12] The video was recorded 136 ms per frame with a total of 96 frames and 100 ms exposure time. Image size was adjusted to show individual nuclei and intensity thresholds were set on the basis of the ratios between nuclear foci signals to background nucleoplasmic fluorescence.

Image Processing

The images were registered and analyzed by *Fiji*[13] and *Mathematica* (Wolfram) software. To achieve subpixel registration accuracy, parameters for shifting, scaling, and rotating camera images were determined by the least-squares fitting of fluorescent bead images (100 nm TetraSpeck fluorescent microspheres, Invitrogen). The experimental data from each channel were processed through an affine transformation and overlapped in false-color channels for visualization. The locus trajectory was obtained by the tracking of locus position over time and graphs were generated by *OriginPro* (OriginLab version 2019b).

S2.12. Additional Data on genomic loci labeling in U2OS cells.

Intensity quantification (Figure S2.2) was performed as follows in equation (2):

$$R_I = \frac{I_F - I_B}{I_N - I_B} \quad (2)$$

Where R_I is the intensity ratio between the labeled IDR3 loci (I_F) and the nucleoplasm (I_N). The background fluorescence intensity (I_B) from a dark region in the same image was subtracted.

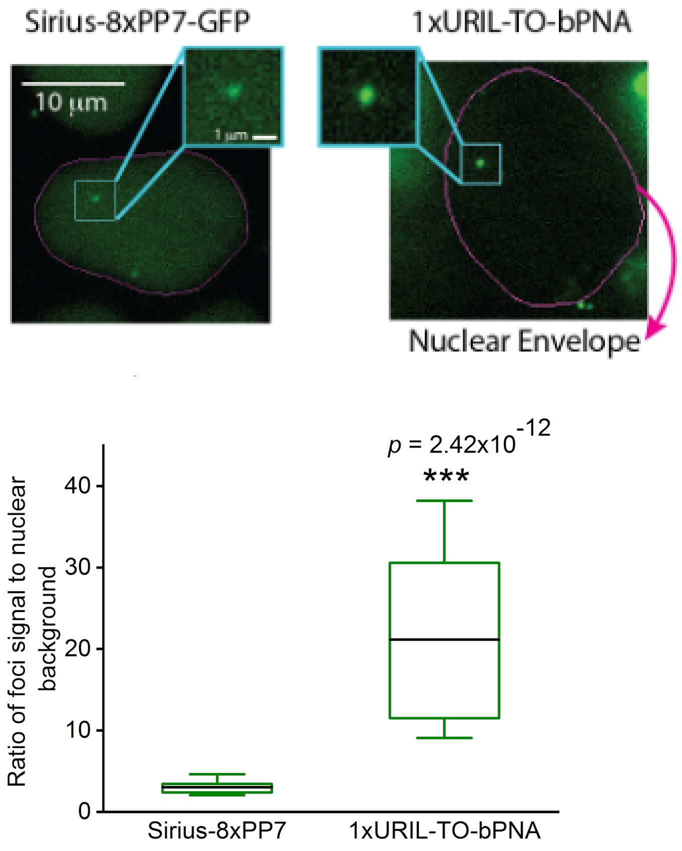

Supplementary Figure 3. High focus-to-nuclear background ratio of 1xURIL-TO-bPNA for DNA imaging. Comparison of the focus-to-nuclear background ratio using CRISPR-Sirius-IDR3-8xPP7/PCP-GFP (sgRNA structure as shown in Fig. 2D) and IDR3-1xURIL-TO-bPNA (sgRNA structure as shown in Fig. 3C and Supplementary Figure 1). The nuclear periphery is outlined in pink. Insets show enlarged images of the genomic loci. (Top row) Images were captured using the same microscope settings and scaled to the same grey levels. All experiments were repeated at least three times (biological replicates). (Bottom) Quantification of the focus/nucleoplasm intensity ratio for the two labeling methods, respectively. The lines within the boxes represent the mean; the outer edges of the box are the 10th and 90th percentiles; the whiskers extend to the minimum and maximum values; n_{cell} = 31 for each experiment. The significance test (two-tailed Welch t-test) was performed at 95% confidence level.

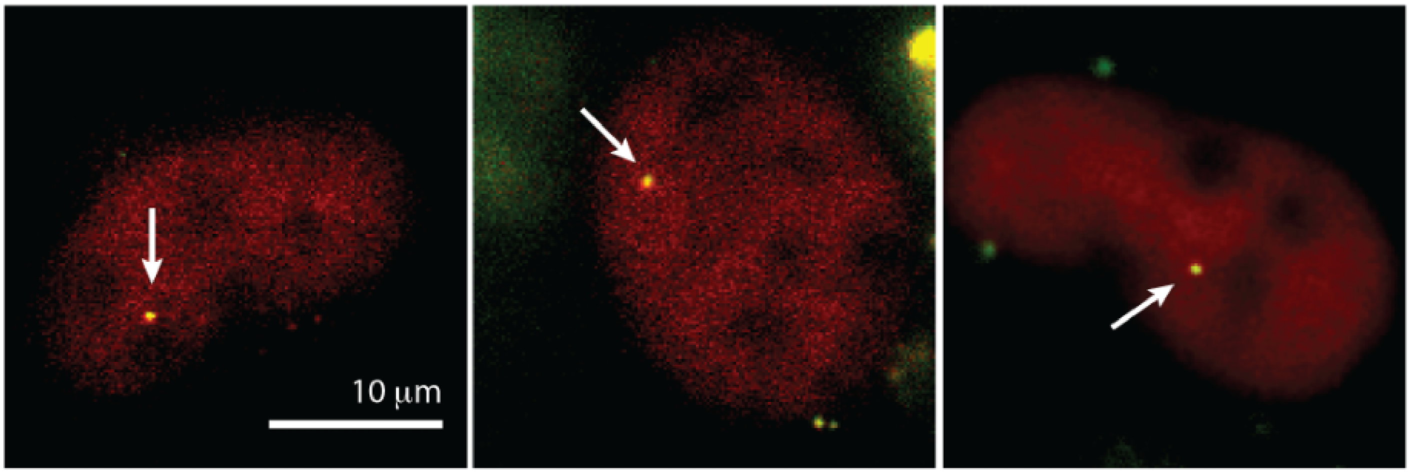

Supplementary Figure 4. Labeling of IDR3 using 1xURIL-TO-bPNA. Additional cell images that show successful labeling of the targeted genomic locus IDR3 (white arrows) in U2OS. Images are overlayed with CRISPR-Sirius-IDR2-8xMS2-Halotag-JF549 for better visualization of the nucleus. Data are representative of experiments performed at least three times.

S3. Additional fluorescence microscopy data (fixed cells)

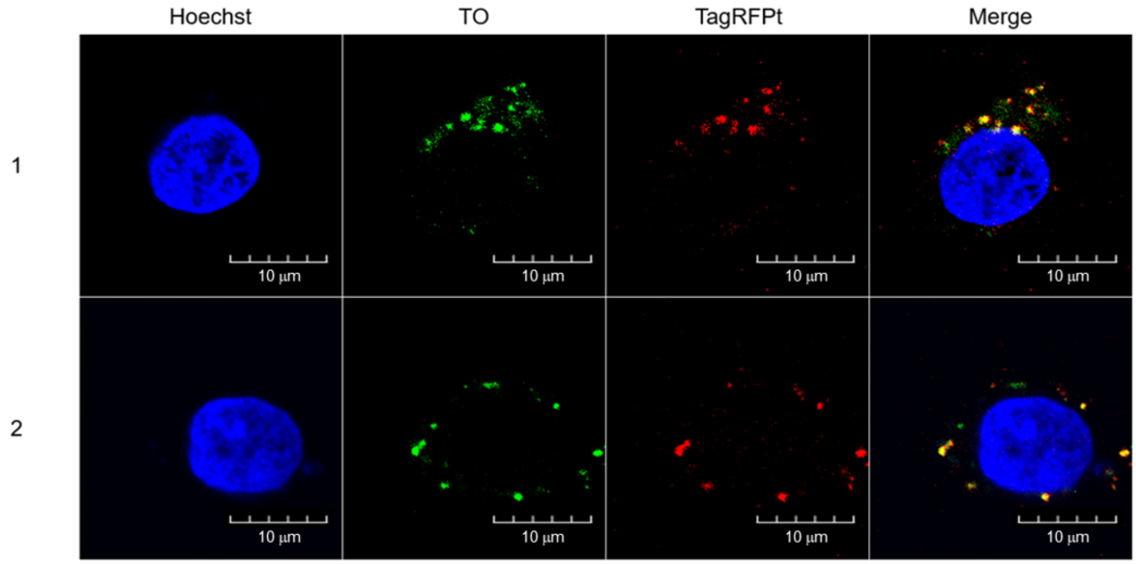

Supplementary Figure 5. Predicted RNA secondary structures for URIL RNA probe constructs.[1–3]. Colocalization of bPNA-TO and MCP-TagRFPt 2 hours after treatment with TO-bPNA. HEK-293 cells were transfected with the plasmids encoding MS2U4-tRNA and MCP-TagRFPt by Lipofectamine 3000. 1 µM K2M-Ala-K2M-TO was added for incubation at 37°C for 2 hours before imaging. Rows 1 and 2 were from two separate treatments; Row 1 is shown in manuscript Figure 5. Triplicate independent experiments yielded similar results.

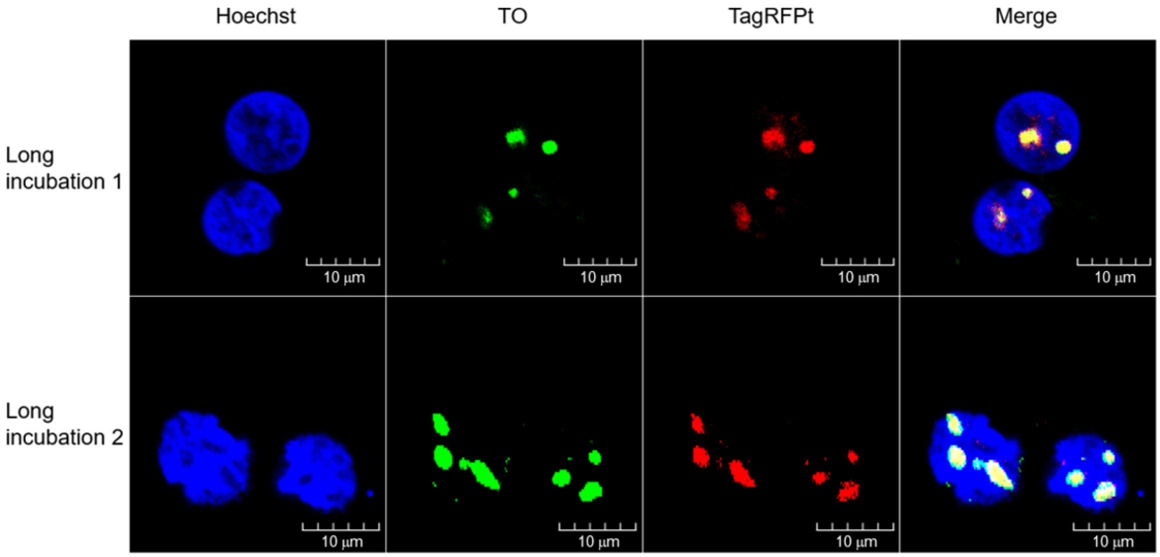

Supplementary Figure 6. Colocalization of bPNA-TO and MCP-TagRFPt 8 hr after treatment with TO-bPNA. HEK-293 cells were treated with MS2U4-tRNA, MCP-TagRFPt and bPNA-TO. Rows 1 and 2 were from two separate treatments; Row 1 is shown in manuscript Figure 5. Triplicate independent experiments yielded similar results.

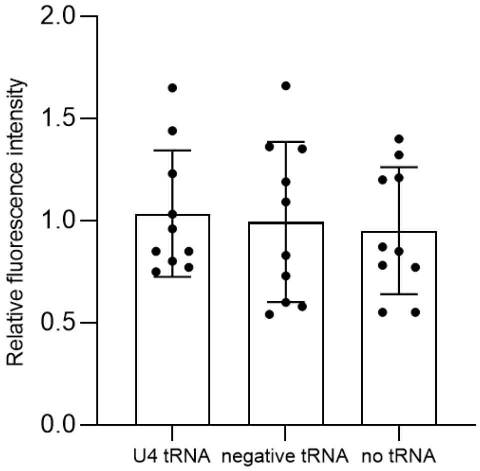

Supplementary Figure 7. Cellular fluorescence intensity following treatment with Cy5-bPNA in place of TO-bPNA. HEK-293 cells were transfected as previously described with U4-tRNA, negative-tRNA or no tRNA. The relative intensities were normalized. Data from 10 independent measurements are shown, with mean values indicated by the top of the bar and standard deviation error shown.

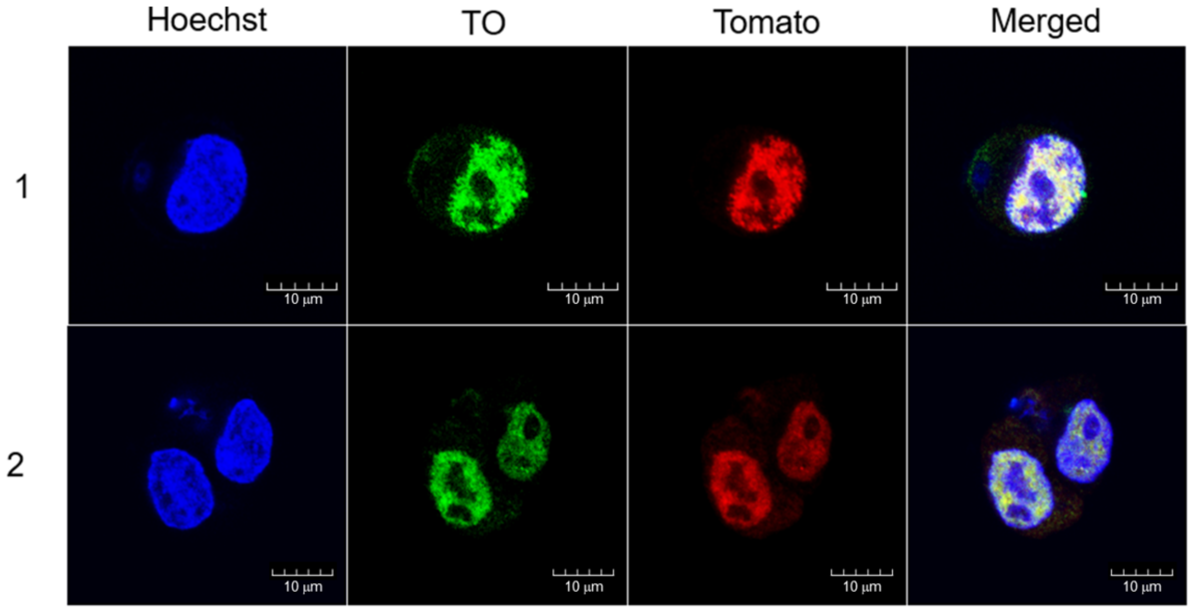

Supplementary Figure 8. Additional replicate showing nuclear colocalization of TO-bPNA and TDP43-tdTomato fluorescence when co-expressed with U4-$(GU)_8$ in HEK-293 cells. Row 2 is shown in Figure 6, manuscript. Triplicate independent experiments yielded similar results.

Supplementary Figure 9. Additional replicates of NEG tRNA transfection and staining with TO-bPNA[a]

NEG-RNA replicate 1 *null labeling*

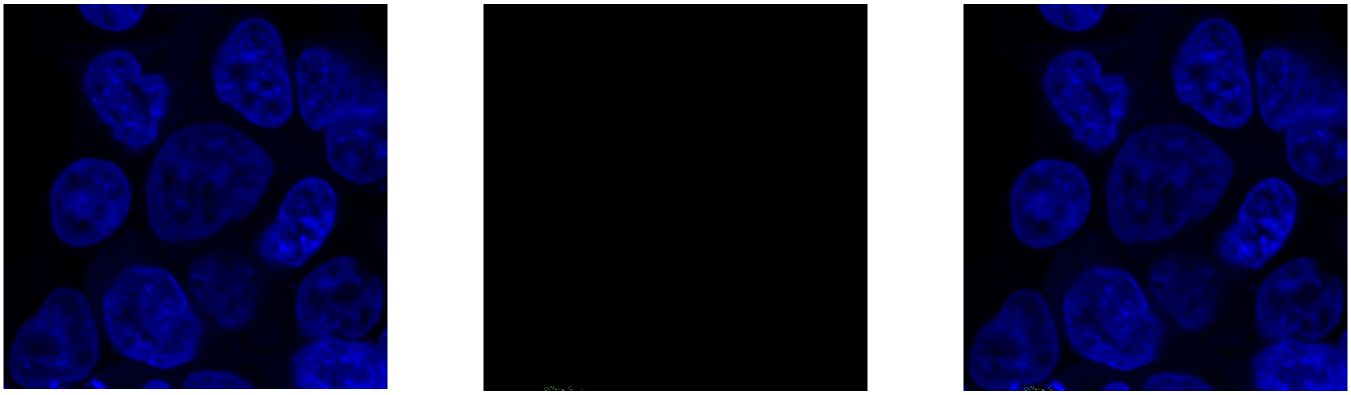

NEG-RNA replicate 2 *null labeling*

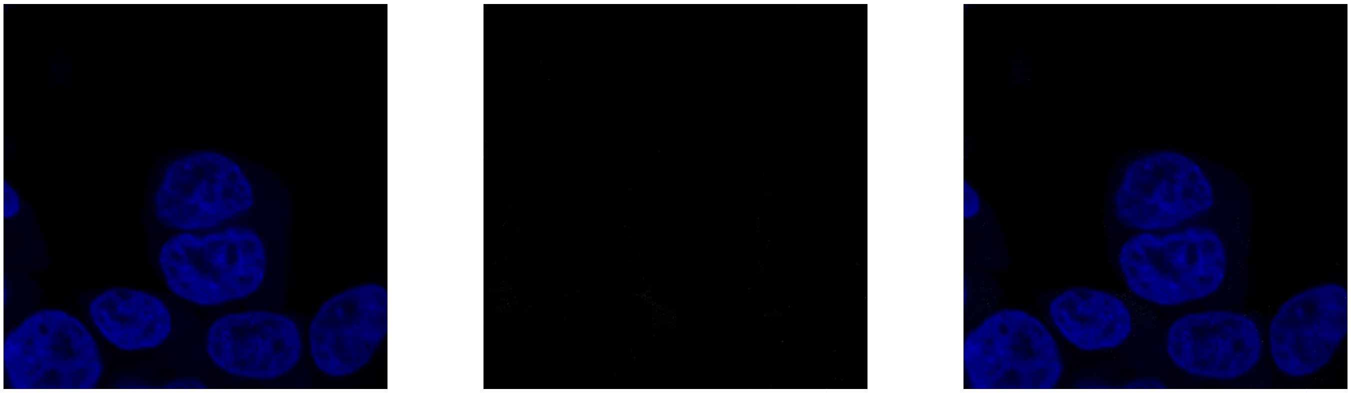

NEG-RNA replicate 3 *null labeling*

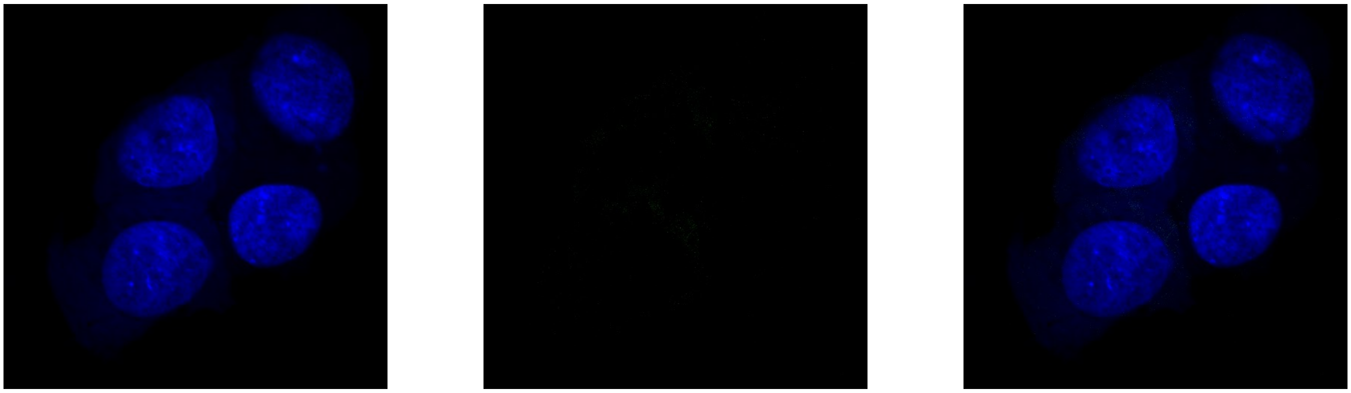

[a]All NEG-RNA and U4-RNA samples run with identical microscopy settings as described. Columns arranged as Hoechst dye channel, green channel and merge channel from left to right.

Supplementary Figure 10. Additional replicates of U4-RNA transfection and staining with TO-bPNA[a]

Hoechst | TO-bPNA | Merge

U4-RNA replicate 1 — *60/62 cells labeled (97%)*

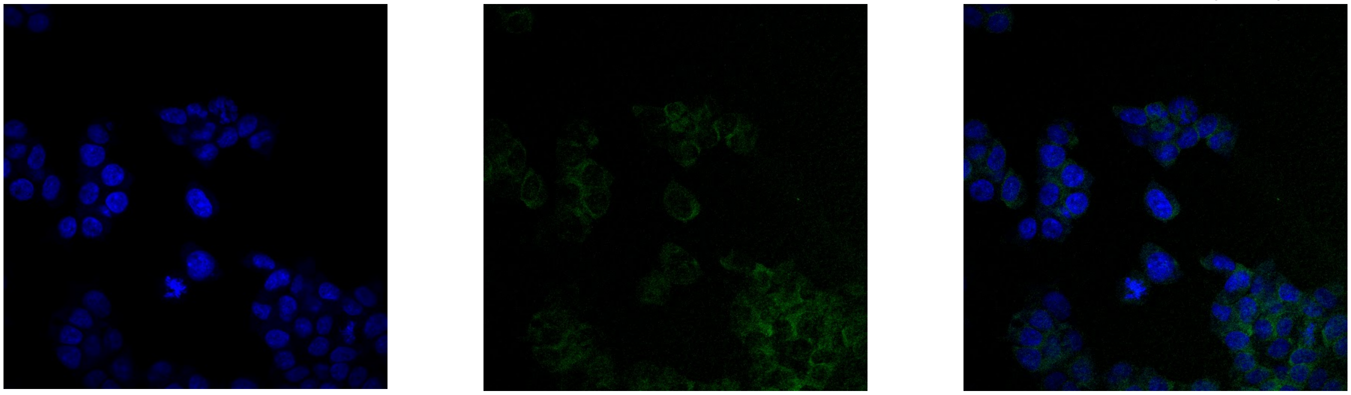

U4-RNA replicate 2 — *20/23 cells labeled (87%)*

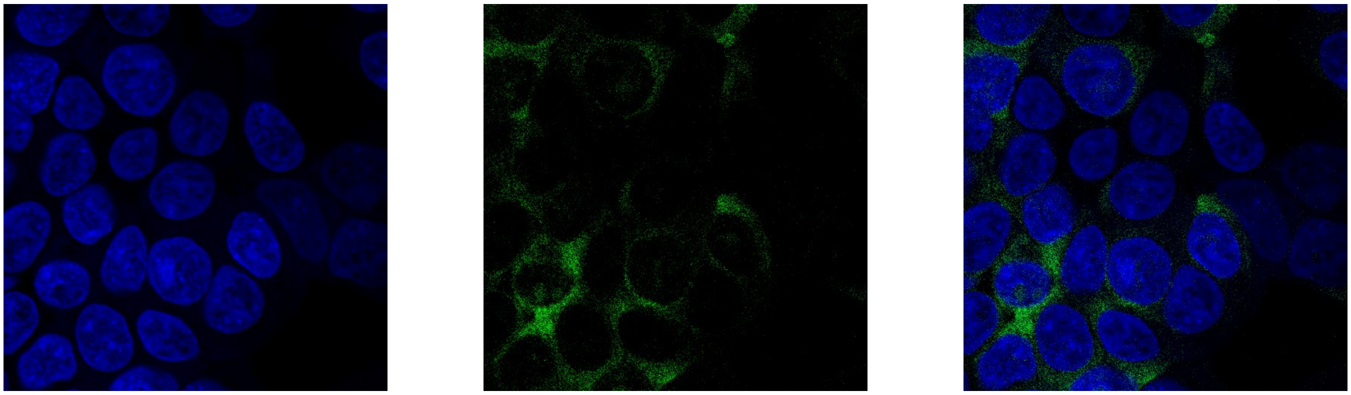

U4-RNA replicate 3 — *98/101 cells labeled (97%)*

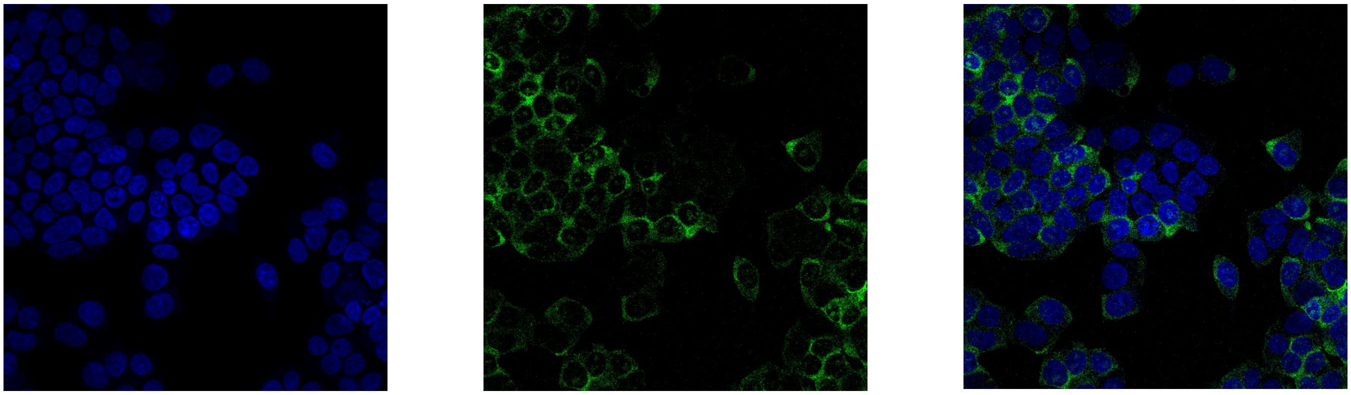

U4-RNA replicate 4 — *146/151 cells labeled (97%)*

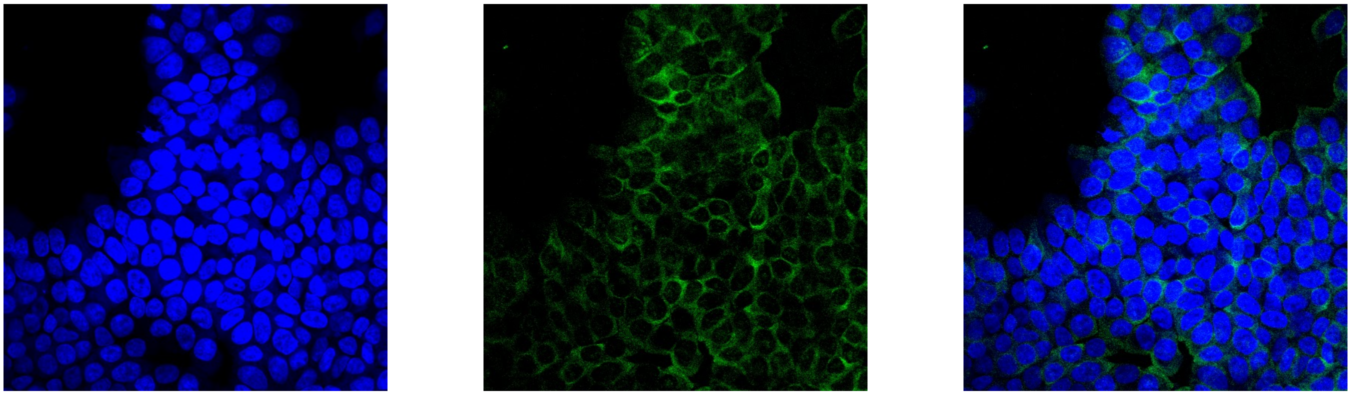

[a]All NEG-RNA and U4-RNA samples run with identical microscopy settings as described. Columns arranged as Hoechst dye channel, green channel and merge channel from left to right.

Supplementary Figure 11. Additional replicates of MCP-RFP colocalization with TO-bPNA with U4-MS2 RNA

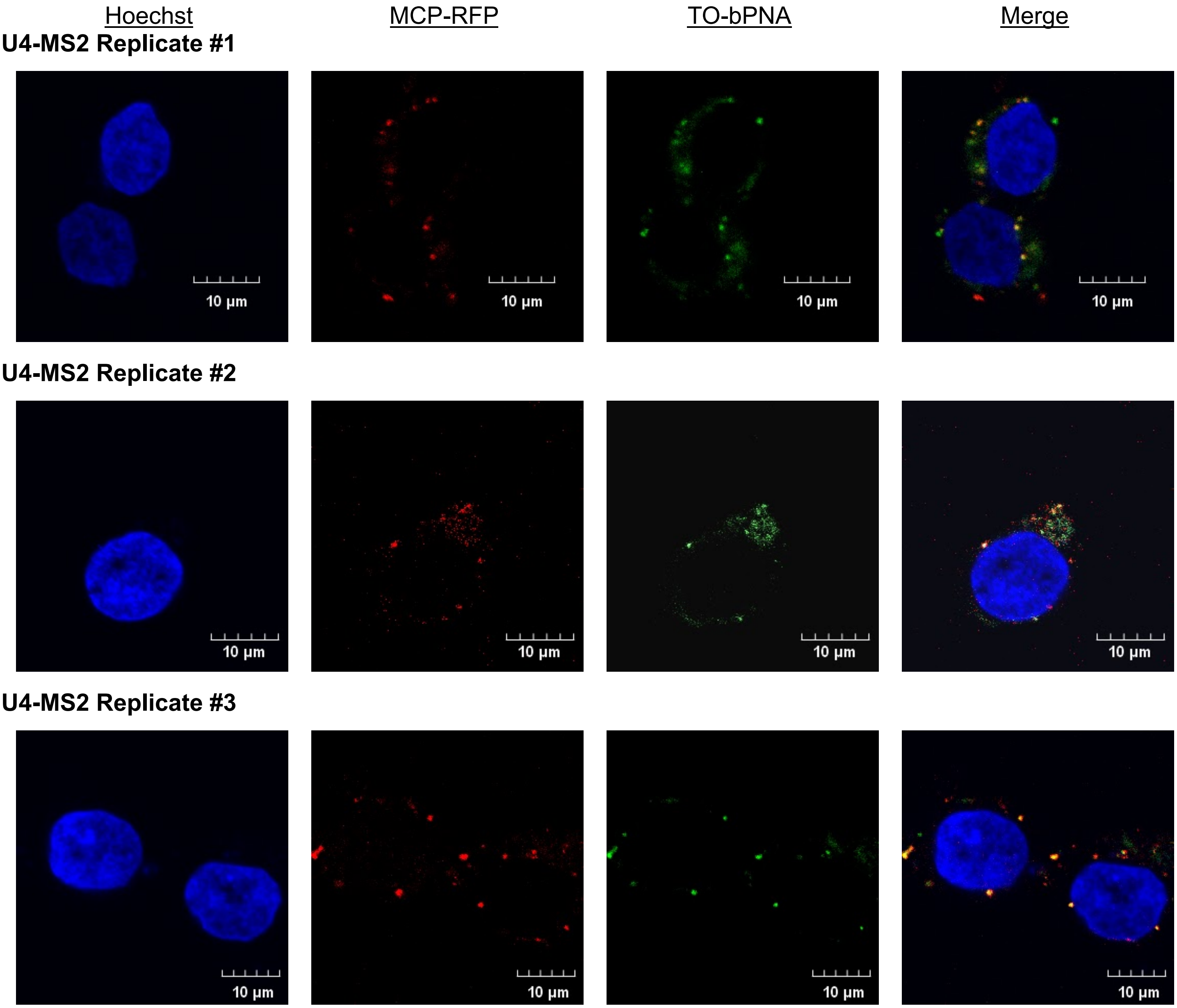

S4. In vitro fluorogenic binding of TO-bPNA with RNAs.

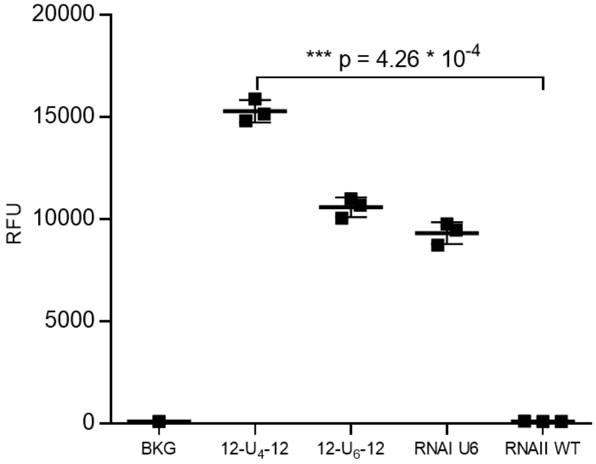

Supplementary Figure 12. Fluorescence turn-on of TO-K^{2M}-Ala-K^{2M} with RNAs as indicated. The mean value of 3 independent replicates with standard deviation error is shown with P-value as indicated, determined using an unpaired, two-tailed, Two-Sample Assuming Unequal Variances t-test.

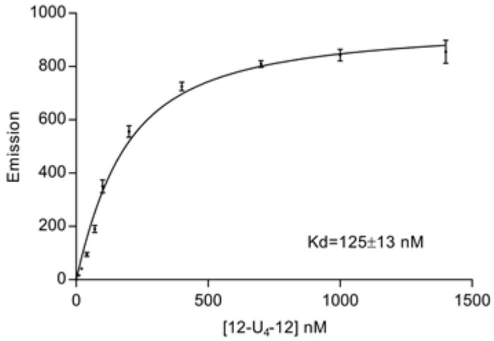

Supplementary Figure 13. Fluorescence activation (apparent K_d) of TO-K2M-Ala-K2M with 12-U_4-12 RNA. Concentration of bPNA is 100 nM. Experimental conditions and fitting as described (S2.3). The mean value of 3 independent replicates is plotted with standard deviation shown in error bars.

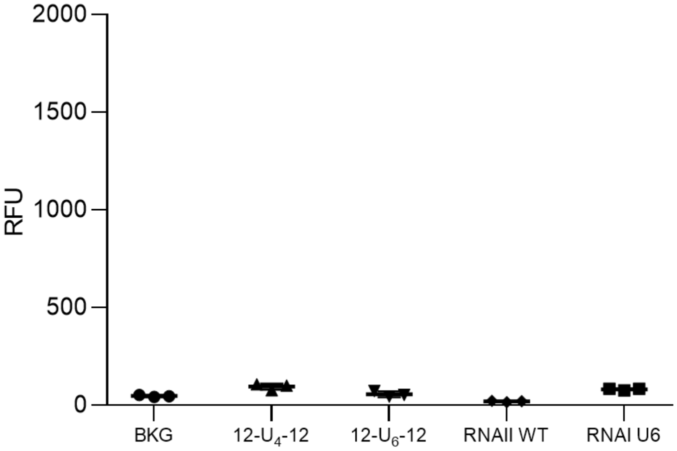

Supplementary Figure 14. Fluorescence of DFHBI-K2M-Ala-K2M upon treatment with RNAs indicated. No fluorescence activation was observed. The mean value of 3 independent replicates with standard deviation error is shown with P-value as indicated.

S5. Synthetic procedures

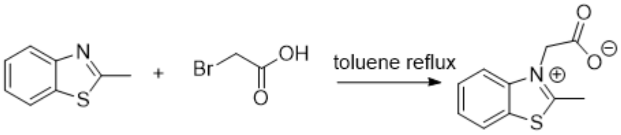

1a 1b 1c

Supplementary Figure 15. Synthesis of 2-(2-Methylbenzo[d]thiazol-3-ium-3-yl)acetate (**1c**).

2-(2-Methylbenzo[d]thiazol-3-ium-3-yl)acetate (2c). The synthesis procedure was adapted from the reported procedure.[14] A mixture of 2-methylbenzothiazole (**1a**, 4.78 g, 32 mmol, 1.0 eq.) and bromoacetic acid (**1b**, 6.5 g, 47 mmol, 1.5 eq.) in toluene (100 ml) was heated at reflux overnight. After cooling to room temperature, the resulting mixture was filtered and the solid was washed with toluene (3 x 5 ml) to afford the product (**1c**, 6.5 g, 98%) after drying.

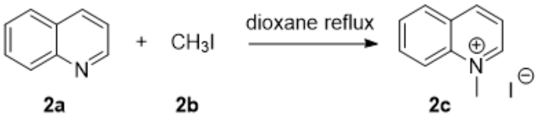

2a 2b 2c

Supplementary Figure 16. Synthesis of 1-methylquinolin-1-ium iodide (**2c**).

1-methylquinolin-1-ium iodide (2c). The synthesis procedure was adapted from the reported procedure.[14] A mixture of quinoline (**2a**, 2 g, 15.5 mmol, 1.0 eq.) and iodomethane (**2b**, 2 ml, 32.2 mmol, 2.1 eq.) in 1,4-dioxane (150 ml) was heated at reflux for 1 hr. After cooling to room temperature, the resulting mixture was filtered and the solid was washed with diethyl ether (3 x 5 ml) and hexanes (3 x 5 ml), affording the product (**2c**, 4 g, 95%) after drying.

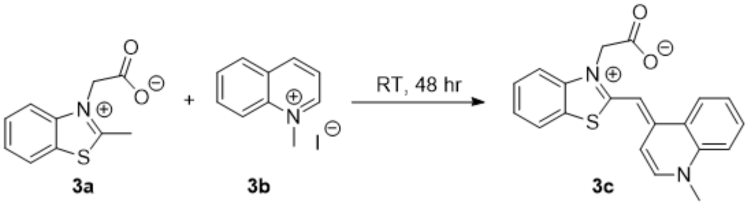

3a 3b 3c

Supplementary Figure 17. Synthesis of TO-acetate (**3c**).

TO-acetate (3c). The synthesis procedure was adapted from the reported procedure.[14] 2-(2-Methylbenzo[d]thiazol-3-ium-3-yl)acetate (**3a**, 2.14 g, 10.4 mmol, 1.4 eq.), 1-methylquinolin-1-ium iodide (**3b**, 2 g, 7.4 mmol, 1 eq.) and triethylamine (2.24 g, 22.2 mmol, 3 eq.) were added in a round bottom flask with 30 ml dichloromethane. The mixture was stirred at room temperature for 48 hr. After 48 hr, the mixture was concentrated and 16 ml ethanol and 4 ml diethyl ether were added. The resulting mixture was stirred at 4 $^{\circ}$C overnight. The resulting precipitate was collected by filtration and washed with diethyl ether (3 x 8 ml). The solid was then dissolved in 12 ml methanol with 3 ml water and was stirred overnight at 4 $^{\circ}$C. The resulting precipitate was collected by filtration and washed with water (3 x 8 ml). Then the solid was resuspended in acetone and stirred at room temperature for 1 hr. The final product was obtained by filtration and was washed with acetone (3 x 8 ml) and dried in vacuum to afford TO-acetate as red solid (**3c**, 160 mg, 6.4%). **^{1}H NMR** (DMSO-d6): 8.54 (1H, d); 8.48 (1H, d); 7.98 (1H, d); 7.94 (1H, d); 7.86 (1H, t); 7.64 (1H, d); 7.55 (2H, m); 7.38 (1H, t); 7.18 (1H, d); 6.77 (1H, s); 5.09 (2H, s); 4.10 (3H, s). **HRMS** (ESI): Mass calculated: [M+H$^+$]=349.1005; Mass found: [M+H$^+$]=349.1004

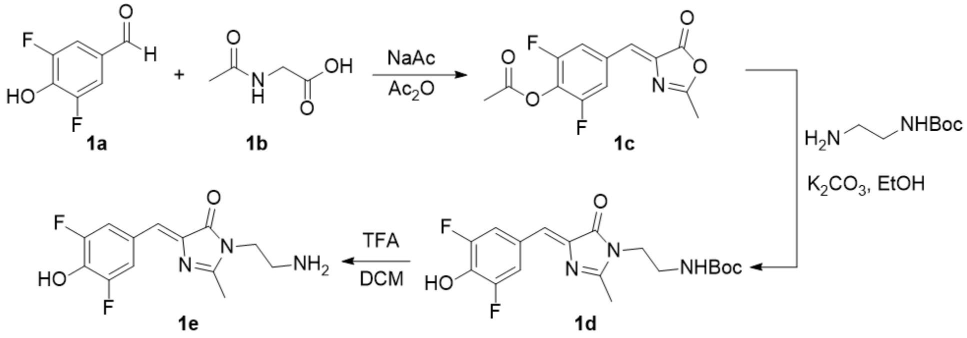

Supplementary Figure 18. Synthesis of (Z)-2,6-difluoro-4-((2-methyl-5-oxo oxazol-4(5H)-ylidene) methyl) phenyl acetate.

(Z)-2,6-difluoro-4-((2-methyl-5-oxooxazol-4(5H)-ylidene)methyl)phenyl acetate (1c). The synthesis was adjusted from a reported procedure.[15] N-Acetylglycine (**1b**, 0.22 g, 1.9 mmol), anhydrous sodium acetate (0.156 g, 1.9 mmol), 4-hydroxy-3,5- difluorobenzaldehyde (**1a**, 0.3 g, 1.9 mmol), and acetic anhydride (0.72 ml) were stirred at 100 ºC for 2 h. Then the reaction was cooled to room temperature and 3 ml ethanol was added to the mixture. The mixture was stirred at 4 ºC overnight. The resulting solid was collected by filtration, washed with cold ethanol, hot water, hexanes and dried to afford 0.32 g (60%) of product as a yellow solid.

DFHBI-ethylenediamine (1e). The synthesis was adjusted from a reported procedure.[15] (Z)-2,6-difluoro-4-((2-methyl-5-oxooxazol-4(5H)-ylidene)methyl)phenyl acetate (**1c**, 100 mg, 0.36 mmol), Boc-ethylenediamine (110 mg, 0.69 mmol) and potassium carbonate (0.13 g) were added to 2 ml ethanol and refluxed for 4 hr. The mixture was cooled to room temperature and the solvent was removed in vacuum. The residue was redissolved in acetate buffer (pH=3.0) and ethyl acetate (1:1 mixture). The organic layer was collected and the solvent was removed in vacuum. The residue was purified by column (DCM:MeOH=10:1), yielding 104 mg (76%) product (**1d**) as yellow solid. The product was added to TFA:DCM=1:1 for Boc deprotection.

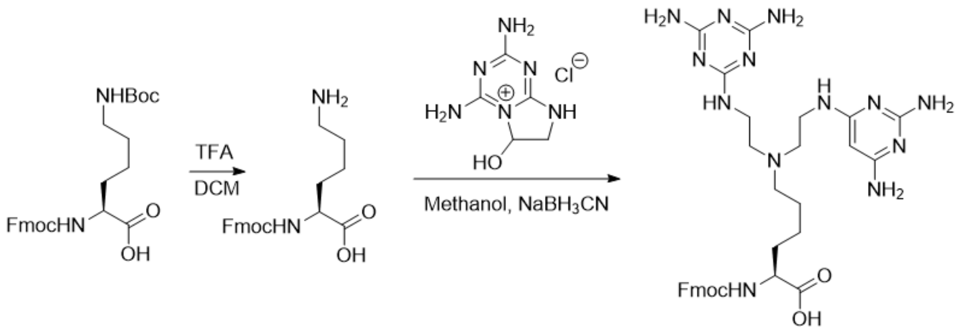

Supplementary Figure 19. Synthesis of Fmoc-K^{2M}-OH.

Fmoc-K^{2M}-OH. The procedure was the same as previously reported.[16] Fmoc-Lys(Boc)-OH (10 g, 21 mmol) was dissolved in 100 mL dichloromethane and 50 mL of trifluoroacetic acid was added. The reaction was stirred for 1 h and the solution was condensed to syrup under a stream of N_2. Dichloromethane (50 mL) was added to dissolve the syrup and was removed by a stream of N_2. The syrup was dissolved in 200 mL methanol and the pH was adjusted to 5 with solid $NaHCO_3$. Melamine aldehyde (Hemiaminal form, 9.5 g, 46.2 mmol) and $NaBH_3CN$ (2.93 g, 46.2 mmol) was aliquoted into 4 portions, respectively. To the reaction solution, 2 portions of melamine aldehyde were added and were stirred and incubated at 50 ºC for 30 min. Then the reaction was taken out to cool to room temperature and 1 portion of $NaBH_3CN$ was added. The reaction was stirred at room temperature for another 30 min. Then 1 portion of aldehyde was added, incubated at 50 ºC for 30 min and 1 portion of $NaBH_3CN$ was added and incubated at room temperature for 30 min. These addition of aldehyde and reductant steps were repeated until all 4 portions of aldehyde and 3 portions of $NaBH_3CN$ were added. The last portion of $NaBH_3CN$ was added to the reaction and stirred for 30 min at room temperature. The reaction was monitored by HPLC. The remaining monoadduct (Fmoc-K1M-OH) was reacted by adding 2.3

g of melamine aldehyde, incubating at 50 ºC for 30 min and 0.72 g $NaBH_3CN$ was added to finish the reaction. Methanol was reduced to ~50 ml and was discarded after centrifugation, yielding white solid as crude product after drying. The reaction was quenched by adding 10 mL 1N hydrochloric acid and the solid was triturated. The hydrochloric acid was discarded after centrifugation. Acetone (30 mL) was added to the residue and the residue was triturated. The acetone was removed by centrifugation. The acetone wash was repeated 3 times and ethanol was added for trituration instead of acetone. After removing ethanol by centrifugation, the product (~11 g, 78%) was obtained as white solid.

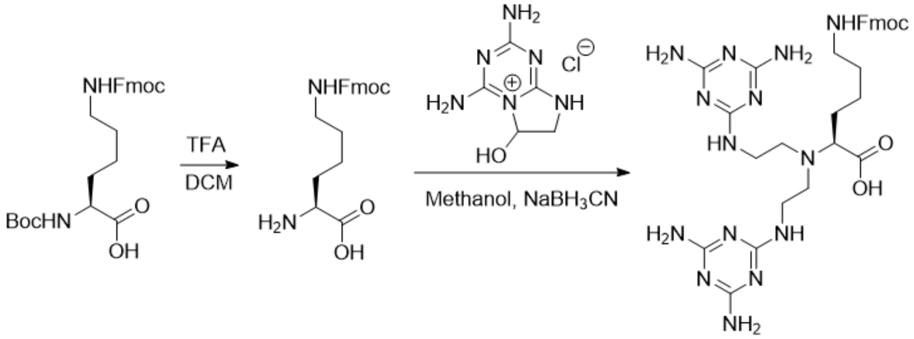

Supplementary Figure 20. Synthesis of Fmoc-αK^{2M}-OH.

Fmoc-αK^{2M}-OH. The procedure was the same as previously reported.[17] Boc-Lys(Fmoc)-OH (1 g, 2.1 mmol) was dissolved in 20 mL dichloromethane and 4 mL of trifluoroacetic acid was added. The reaction was stirred for 1 h and the solution was condensed to syrup under a stream of N_2. Dichloromethane (10 mL) was added to dissolve the syrup and was removed by a stream of N_2. The syrup was dissolved in 30 mL methanol and the pH was adjusted to 5 with solid $NaHCO_3$. Melamine aldehyde (0.95 g, 4.62 mmol) and $NaBH_3CN$ (0.293 g, 4.62 mmol) was aliquoted into 4 portions, respectively. To the reaction solution, 2 portions of melamine aldehyde were added and incubated at 50˚C for 30 min with stirring. Then the reaction was taken out to cool to room temperature and 1 portion of $NaBH_3CN$ was added. The reaction was stirred at room temperature for another 40 min. Then 1 portion of aldehyde was added, incubated at 50˚C for 30 min and 1 portion of $NaBH_3CN$ was added and incubated at room temperature for 40 min. These additions of aldehyde and reductant steps were repeated until all 4 portions of aldehyde and 3 portions of $NaBH_3CN$ were added. The last portion of $NaBH_3CN$ was added to the reaction and stirred for 40 min at room temperature. The reaction was monitored by HPLC. The remaining monoadduct (Fmoc-αK^M-OH) was reacted by adding 0.25 g of melamine aldehyde, incubating at 50˚C for 30 min and 0.078 g $NaBH_3CN$ was added to finish the reaction. Methanol was reduced to 5 ml and was discarded after centrifugation, yielding pale yellow solid as crude product after drying. The reaction was quenched by adding 2 mL 1N hydrochloric acid and the solid was triturated. The hydrochloric acid was discarded after centrifugation. Acetone (5 mL) was added to the residue and the residue was triturated. The acetone was removed by centrifugation. The acetone wash was repeated 3 times and ethanol was added for trituration instead of acetone. After removing ethanol by centrifugation, the product (0.85 g, 60%) was obtained as pale yellow solid. **^{1}H NMR** (400 MHz, DMSO-d6): 7.91 (2H, d); 7.79 (10H, d); 7.66 (2H, d); 7.41 (2H, t); 7.32 (2H, t); 7.25 (1H, t); 4.28 (2H, d); 4.20 (1H, t); 3.33 (4H, q); 3.28 (1H, m); 2.95 (4H, q); 2.76 (2H, q); 1.62 (2H, q); 1.15-1.50 (4H, m). **^{13}C NMR** (100 MHz, DMSO): 174.3; 170.7; 166.3; 154.5; 144.3; 141.1; 128.0; 127.5; 125.5; 120.5; 80.1; 79.6; 65.5; 50.6; 49.0; 47.2; 29.3; 23.6; 21.9. **HRMS** (ESI): calculated for [M+H]=673.3430, [M+2H]=337.1751, found [M+H]=673.3425, [M+2H]=337.1756.

S6. Solid phase peptide synthesis

Peptide synthesis was performed manually using Rink Amide resin (100-200 mesh, loading 0.3 mmol/g) employing standard Fmoc chemistry. With 150 mg resin, 0.25 M of Amino acids were coupled with 0.25 M of PyAOP and 0.25 M DIPEA in 2 ml NMP. Fluorogenic dyes were coupled using three equivalents of dye, 3.3 equivalents of HBTU, and 3.3 equivalents of DIPEA in 2 ml DMF. Fmoc cleavage was performed with 2 mL of piperidine:NMP (1:1) with 3% DBU. Dye and coupling reagents were allowed to react for 15 min before addition to resin. For TO peptides, TO- acetate was coupled to the peptide N-terminus directly. For DFHBI peptide, the peptide was reacted with 3 equivalents of succinic anhydride, and 3 equivalents of DIPEA for 15 min,washed and DFHBI-NH_2 was coupled to the N-terminus of the peptide. Peptides were cleaved from the solid support using 95% trifluoroacetic acid (TFA) and 5% H_2O for 2 h. Cold diethyl ether (Et_2O) was added to precipitate the peptide and the crude pellet was washed with cold Et_2O two times and dried over vacuum. Crude peptides were then dissolved in solvent A and purified by HPLC on a semi-prep C_{18} reversed phase column at 8 mL/min. The UV detector was set at 238 nm. The purified peptides were lyophilized to dryness. The identity of peptide was checked by MALDI-TOF and purity checked by analytical HPLC on a C_{18} column. (solvent A=0.1%TFA in water, solvent B=0.07% TFA in 90% acetonitrile, 10% water).

S7 Compound characterization.

- **TO-acetate**

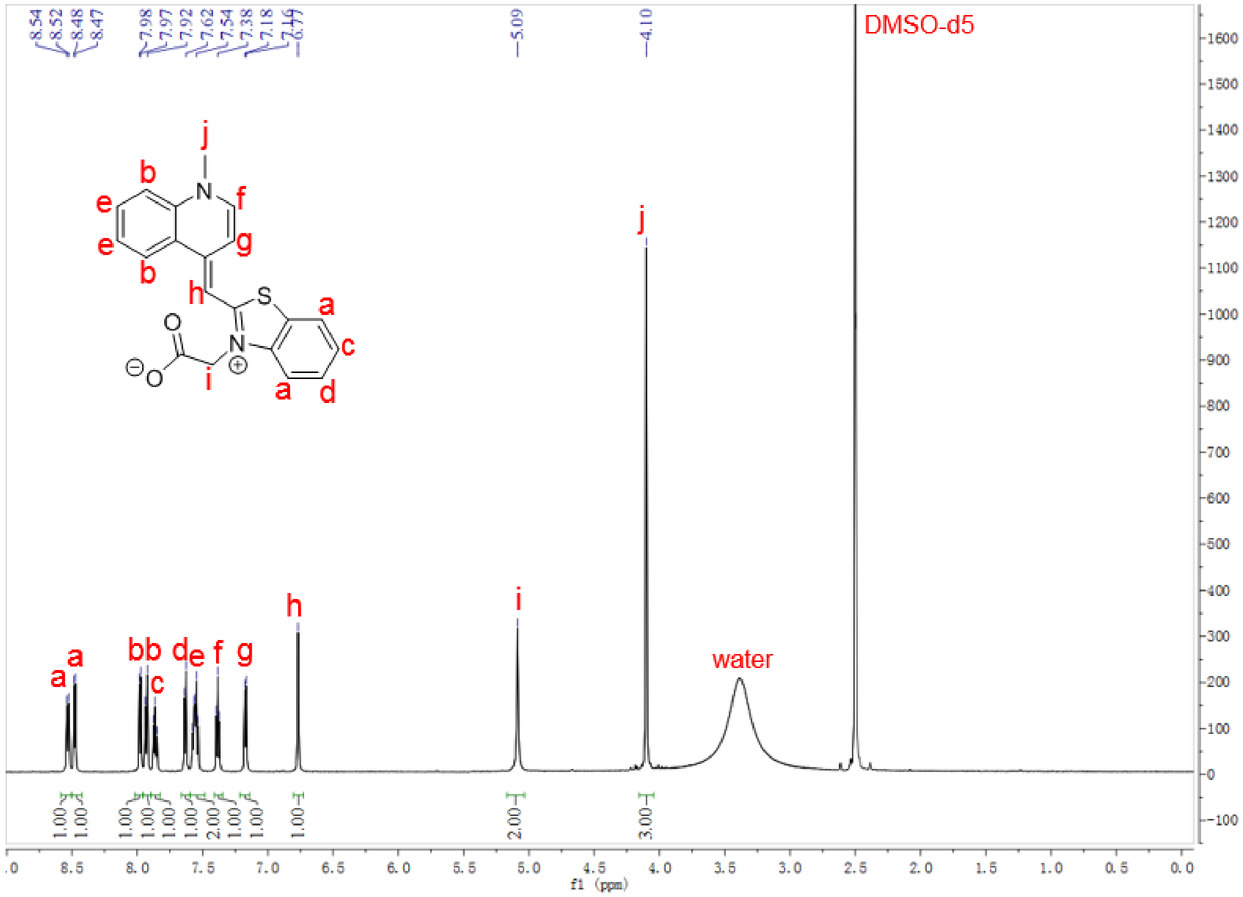

Supplementary Figure 21. ^{1}H NMR of TO-acetate. (DMSO-d6)

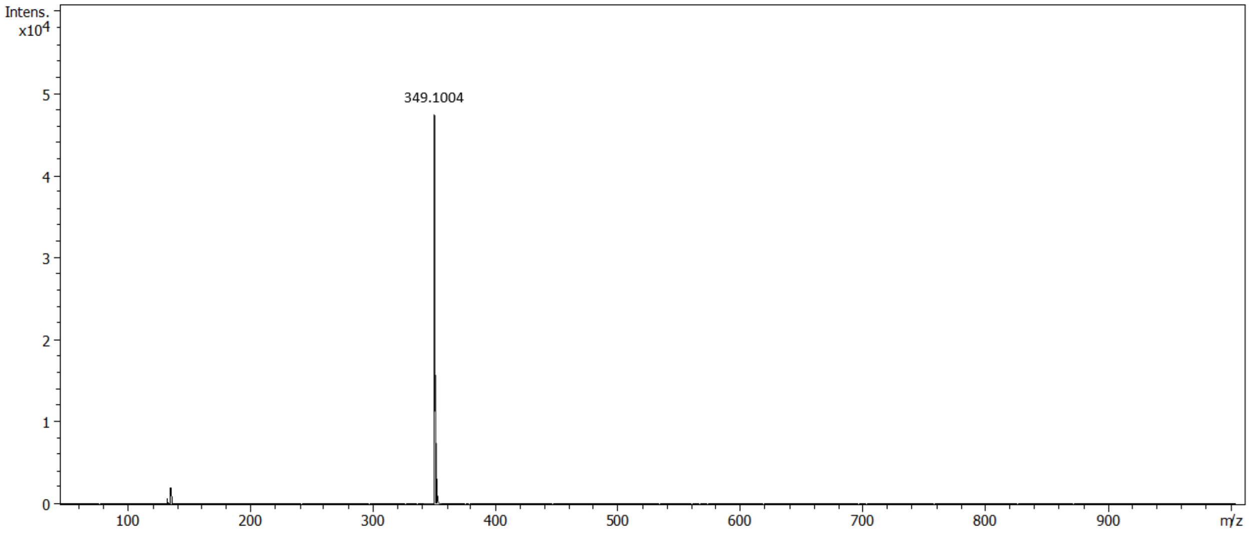

Supplementary Figure 22. ESI of TO-acetate. Mass expt: $[M+H^+]$=349.1004; Mass calc: $[M+H^+]$=349.1005.

- **DFHBI-ethylenediamine-Boc**

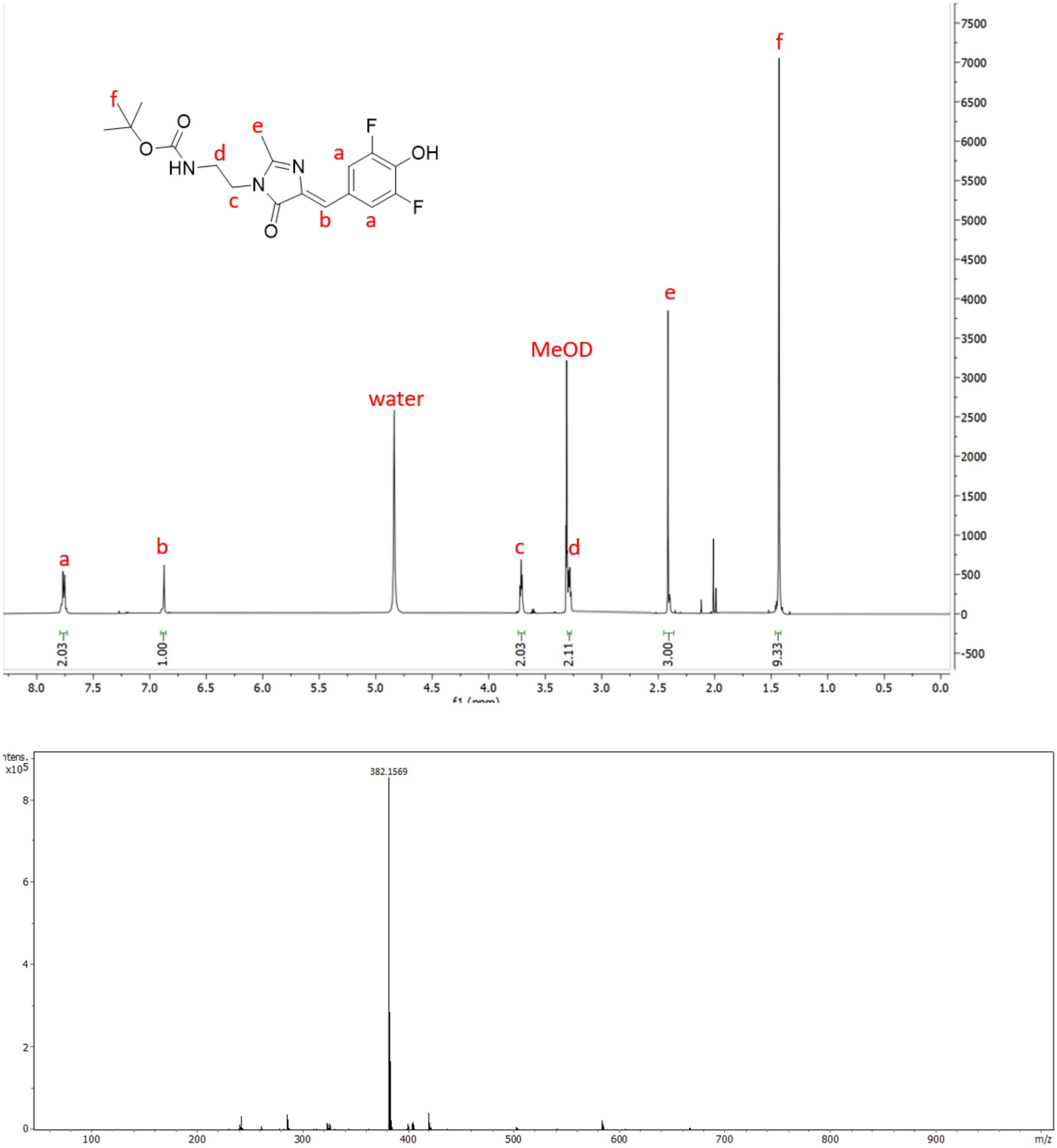

Supplementary Figure 23. (Top) ^{1}H NMR of DFHBI-ethylenediamine-Boc. (DMSO-d6)
(Bottom) ESI of DFHBI-ethylenediamine-Boc. Mass expt: $[M+H^+]$=382.1569; Mass calc: $[M+H^+]$=382.1573.

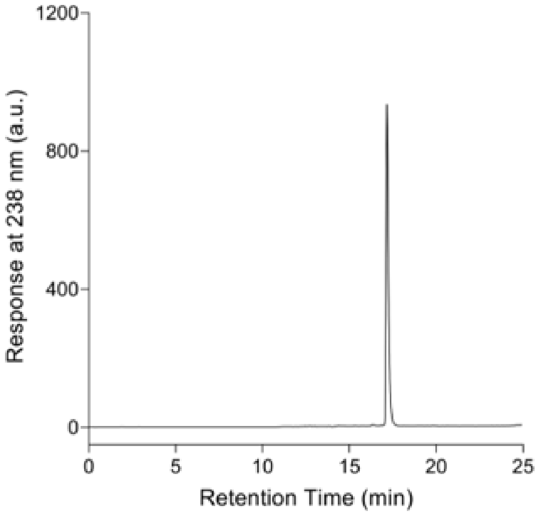

Supplementary Figure 24. HPLC of **4** (K^{2M}-K^{TO}-K^{2M}). Gradient: 0-5 min: 0% B; 5-15 min: 0-40% B; 15-20 min: 40% B; 20-20.5 min: 40-100% B; 20.5-22.5 min: 100% B; 22.5-23 min: 100-0% B; 23-25 min: 0% B.

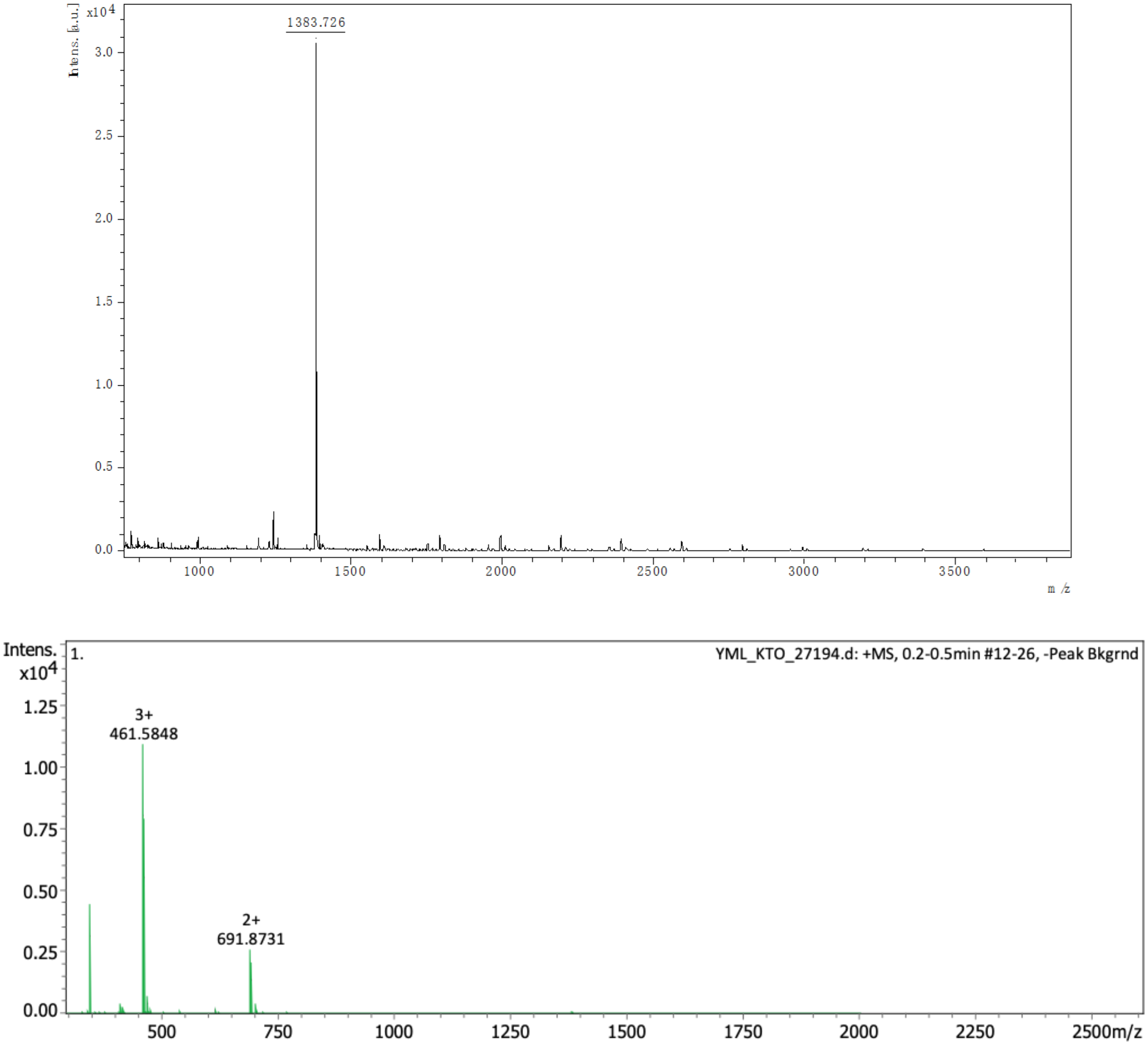

Supplementary Figure 25. (Top) MALDI-TOF of **4** (Ac-K^{2M}-K^{TO}-K^{2M}). Mass calculated: [M+H$^+$]=1383.743; Mass found: [M+H$^+$]=1383.726. (Bottom) High-res electrospray mass spec showing triply and doubly charged ion.

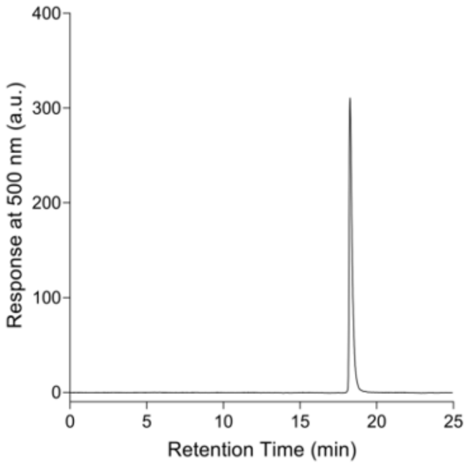

Supplementary Figure 26. HPLC of **1** (TO-βAla-K^{2M}-Ala-K^{2M}) Gradient: 0-5 min: 0% B; 5-15 min: 0-40% B; 15-20 min: 40% B; 20-20.5 min: 40-100% B; 20.5-22.5 min: 100% B; 22.5-23 min: 100-0% B; 23-25 min: 0% B.

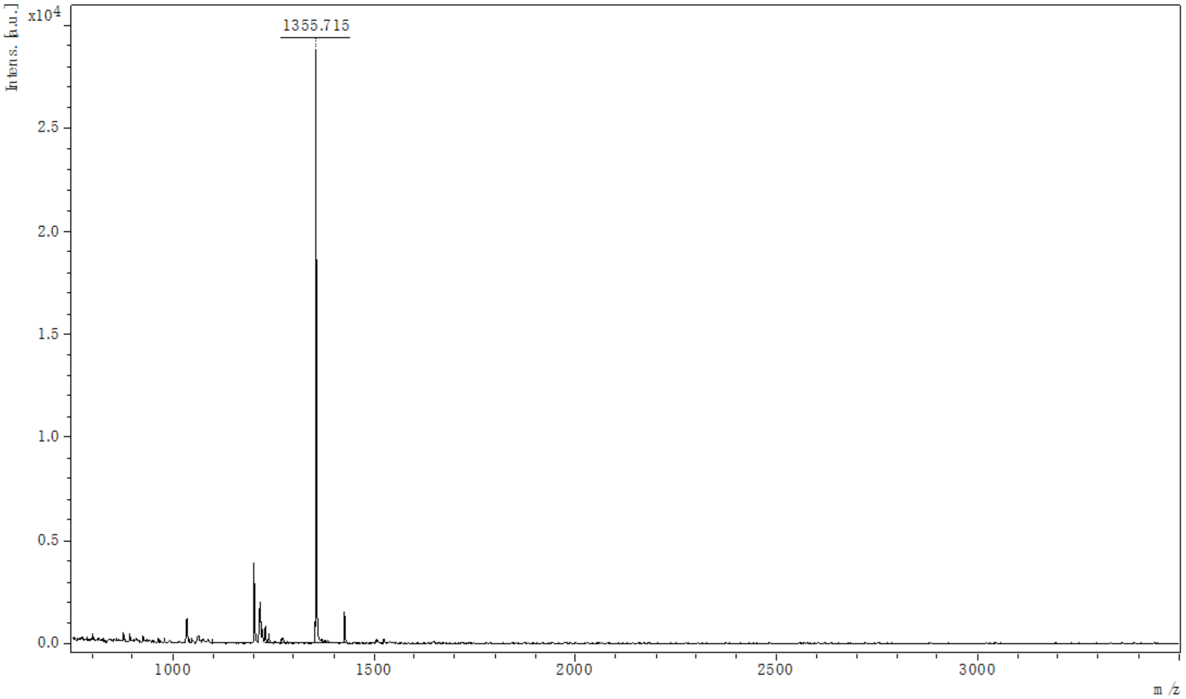

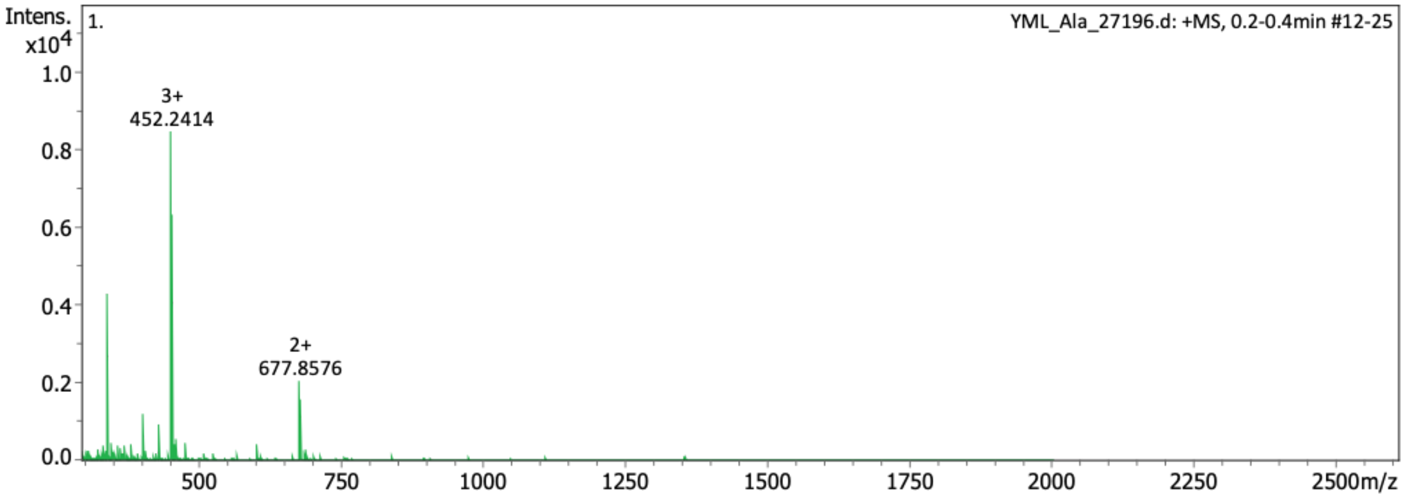

Supplementary Figure 27. (Top) MALDI-TOF of **1** (TO-βAla-K^{2M}-Ala-K^{2M}). (Bottom) High-res electrospray mass spec showing triply and doubly charged ion. Mass calculated: $[M+H^{+}]$=1355.7126; Mass found: MALDI $[M+H^{+}]$=1355.715; ESI [3+]=452.2414 (1356.7242); [2+]=677.8576 (1355.7152).

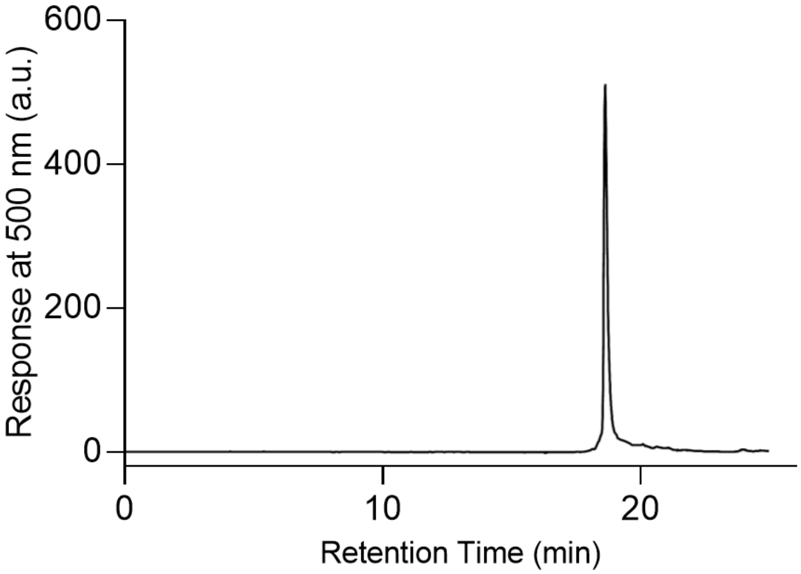

Supplementary Figure 28. HPLC of **2**, TO-βAla-K^{2M}-Ile-K^{2M}. Gradient: 0-5 min: 0% B; 5-15 min: 0-40% B; 15-20 min: 40% B; 20-20.5 min: 40-100% B; 20.5-22.5 min: 100% B; 22.5-23 min: 100-0% B; 23-25 min: 0% B.

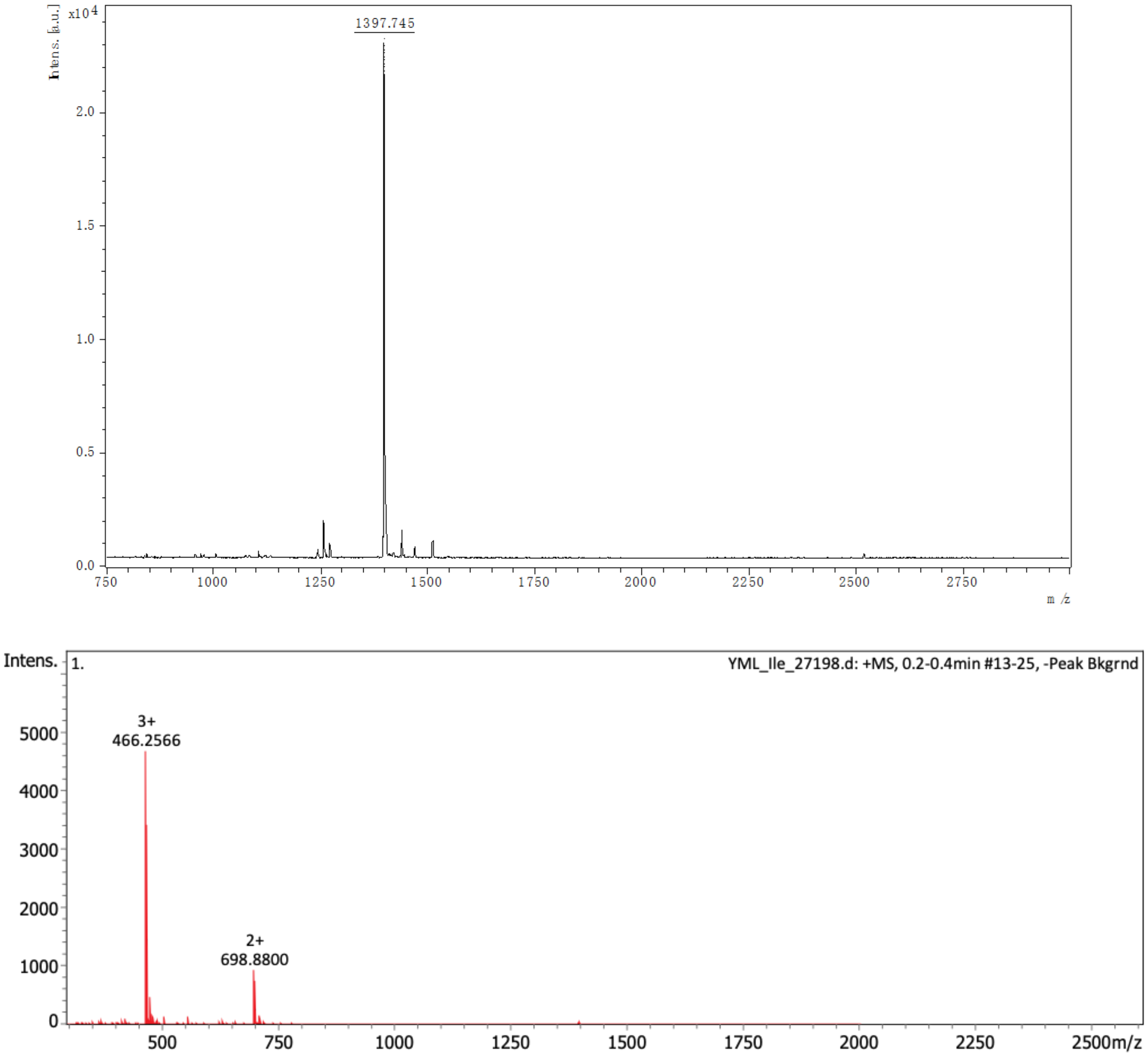

Supplementary Figure 29. (Top) MALDI-TOF of **2**, TO-βAla-K^{2M}-Ile-K^{2M}. (Bottom) High-res electrospray mass spec showing triply and doubly charged ion. Mass calculated: [M+H^{+}]=1397.7596; Mass found: MALDI [M+H^{+}]=1397.745; ESI [3+]=466.2566 (1398.7698), [2+]=698.8800 (1397.7600).

REFERENCES

(1) Lorenz, R.; Bernhart, S. H.; Höner Zu Siederdissen, C.; Tafer, H.; Flamm, C.; Stadler, P. F.; Hofacker, I. L. ViennaRNA Package 2.0. *Algorithms Mol. Biol.* **2011**, *6*, 26.

(2) Gruber, A. R.; Lorenz, R.; Bernhart, S. H.; Neuböck, R.; Hofacker, I. L. The Vienna RNA Websuite. *Nucleic Acids Res.* **2008**, *36* (suppl_2), W70–W74.

(3) Mathews, D. H.; Disney, M. D.; Childs, J. L.; Schroeder, S. J.; Zuker, M.; Turner, D. H. Incorporating Chemical Modification Constraints into a Dynamic Programming Algorithm for Prediction of RNA Secondary Structure. *Proceedings of the National Academy of Sciences* **2004**, *101* (19), 7287–7292.

(4) Brouwer, A. M. Standards for photoluminescence quantum yield measurements in solution (IUPAC Technical Report). *J. Macromol. Sci. Part A Pure Appl. Chem.* **2011**, *83* (12), 2213–2228.

(5) Levitus, M. Tutorial: Measurement of Fluorescence Spectra and Determination of Relative Fluorescence Quantum Yields of Transparent Samples. *Methods Appl Fluoresc* **2020**, *8* (3), 033001.

(6) Song, W.; Filonov, G. S.; Kim, H.; Hirsch, M.; Li, X.; Moon, J. D.; Jaffrey, S. R. Imaging RNA Polymerase III Transcription Using a Photostable RNA-Fluorophore Complex. *Nat. Chem. Biol.* **2017**, *13* (11), 1187–1194.

(7) Yang, C.; Tan, W.; Whittle, C.; Qiu, L.; Cao, L.; Akbarian, S.; Xu, Z. The C-Terminal TDP-43 Fragments Have a High Aggregation Propensity and Harm Neurons by a Dominant-Negative Mechanism. *PLoS One* **2010**, *5* (12), e15878.

(8) Halstead, J. M.; Lionnet, T.; Wilbertz, J. H.; Wippich, F.; Ephrussi, A.; Singer, R. H.; Chao, J. A. An RNA Biosensor for Imaging the First Round of Translation from Single Cells to Living Animals. *Science* **2015**. https://doi.org/10.1126/science.aaa3380.

(9) Ma, H.; Tu, L.-C.; Naseri, A.; Chung, Y.-C.; Grunwald, D.; Zhang, S.; Pederson, T. CRISPR-Sirius: RNA Scaffolds for Signal Amplification in Genome Imaging. *Nat. Methods* **2018**, *15* (11), 928–931.

(10) Ma, H.; Tu, L.-C.; Naseri, A.; Huisman, M.; Zhang, S.; Grunwald, D.; Pederson, T. Multiplexed Labeling of Genomic Loci with dCas9 and Engineered sgRNAs Using CRISPRainbow. *Nat. Biotechnol.* **2016**, *34* (5), 528–530.

(11) Ma, H.; Tu, L.-C.; Chung, Y.-C.; Naseri, A.; Grunwald, D.; Zhang, S.; Pederson, T. Cell Cycle- and Genomic Distance-Dependent Dynamics of a Discrete Chromosomal Region. *J. Cell Biol.* **2019**, *218* (5), 1467–1477.

(12) Chung, Y.-C.; Bisht, M.; Tu, L.-C. CRISPR-Based Multi-Locus Real-Time Tracking Reveals Single Chromosome Dynamics and Compaction. *bioRxiv*, 2022, 2022.02.01.478681. https://doi.org/10.1101/2022.02.01.478681.

(13) Schindelin, J.; Arganda-Carreras, I.; Frise, E.; Kaynig, V.; Longair, M.; Pietzsch, T.; Preibisch, S.; Rueden, C.; Saalfeld, S.; Schmid, B.; Tinevez, J.-Y.; White, D. J.; Hartenstein, V.; Eliceiri, K.; Tomancak, P.; Cardona, A. Fiji: An Open-Source Platform for Biological-Image Analysis. *Nat. Methods* **2012**, *9* (7), 676–682.

(14) Dolgosheina, E. V.; Jeng, S. C. Y.; Panchapakesan, S. S. S.; Cojocaru, R.; Chen, P. S. K.; Wilson, P. D.; Hawkins, N.; Wiggins, P. A.; Unrau, P. J. RNA Mango Aptamer-Fluorophore: A Bright, High-Affinity Complex for RNA Labeling and Tracking. *ACS Chem. Biol.* **2014**, *9* (10), 2412–2420.

(15) Song, W.; Strack, R. L.; Svensen, N.; Jaffrey, S. R. Plug-and-Play Fluorophores Extend the Spectral Properties of Spinach. *J. Am. Chem. Soc.* **2014**, *136* (4), 1198–1201.

(16) Miao, S.; Liang, Y.; Marathe, I.; Mao, J.; DeSantis, C.; Bong, D. Duplex Stem Replacement with bPNA+ Triplex Hybrid Stems Enables Reporting on Tertiary Interactions of Internal RNA Domains. *J. Am. Chem. Soc.* **2019**, *141* (23), 9365–9372.

(17) Miao, S.; Bhunia, D.; Devari, S.; Liang, Y.; Munyaradzi, O.; Rundell, S.; Bong, D. Bifacial PNAs Destabilize MALAT1 by 3’ A-Tail Displacement from the U-Rich Internal Loop. *ACS Chem. Biol.* **2021**, *16* (8), 1600–1609.
